# Synthesis of β-arylated alkylamides via Pd-catalyzed one-pot installation of a directing group and C(sp^3^)–H arylation

**DOI:** 10.3762/bjoc.12.108

**Published:** 2016-06-03

**Authors:** Yunyun Liu, Yi Zhang, Xiaoji Cao, Jie-Ping Wan

**Affiliations:** 1 College of Chemistry and Chemical Engineering, Jiangxi Normal University, Nanchang 330022 P. R. China; 2 Research Center of Analysis and Measurement, Zhejiang University of Technology, 18 Chaowang Road, Hangzhou, Zhejiang 310014, P.R. China

**Keywords:** alkylamides, C–H arylation, directing group, in situ installation, one-pot

## Abstract

The synthesis of β-arylated alkylamides via alkyl C–H bond arylation has been realized by means of direct one-pot reactions of acyl chlorides, aryl iodides and 8-aminoquinoline. Depending on the structure of the starting materials, both single and double β-arylated alkylamides could be accessed.

## Introduction

The elaboration of inert C–H bonds is regarded as the longstanding aim of modern organic synthesis. Upon the extensive efforts during the past decades, the exploration and application of C–H activation/functionalization has won splendid success [1–6]. Among the numerous elegant examples of C–H activation reactions, the DG (directing group) assisted C–H activation is obviously the most generally applicable tactic because of the irreplaceable function of the DG in facilitating the incorporation of a metal catalyst and controlling the site selectivity [7–9]. While benefiting the advantage of straightforward transformation from the C–H activation strategy, the utilization of a DG also brings unfavorable defection of step economics because an additional operation step in installing the DG to reactants is required in most of presently known DG-assisted reactions. For example, in the widely studied reactions employing AQ (8-aminoquinoline) as DG, the prior reaction (including isolation) of the corresponding acyl chlorides and AQ is generally the mandatory procedure before conducting subsequent C–H transformation [10–12]. The additional time in running the DG installation reaction and purification as well as related consumption of chemicals substantially undermine the efficiency of the C–H activation-based synthesis, which is against the principle of step economy [13–14]. In this context, developing alternative strategies which are able to skip the DG-installing procedure is of high emergence in the chemistry of DG-based C–H activation.

To solve the problem of additional cost resulted from the DG installation, the concept of domino reaction in which the multiple chemical bond transformations complete in one-step operation is theoretically the best option [15–20]. On the other hand, owing to their close relevance to biological processes and clinical pharmaceuticals [21–23], the amides constitute a class of most important targets of organic synthesis [24–27]. As a representative protocol for the synthesis of C–H elaborated alkyl/arylamides, the AQ-assisted C–H functionalization reactions have been broadly investigated and applied [28–34]. Since the prior preparation and purification of *N*-AQ amides have been required in all these known syntheses, a domino process by which the AQ can be linked directly to the raw substrates to enable the subsequent arylation transformation in one pot would be highly favorable for enhancing step economy.

Upon this assumption as well as our interest in both domino reactions and C–H functionalization chemistry [35–40], we have executed efforts to the AQ-assisted β-C–H functionalization reactions of alkylamides via activation of the C(sp^3^)–H bond, a classical protocol toward β-arylamide synthesis. While the known examples, including C(sp^3^)–H alkylation, arylation or oxygenation employing different transition metal catalysts such as palladium, nickel, and iron have provided enriched routes for the synthesis of structurally diverse amides, a two step process involving the operation in installing the AQ to the substrate has been required [41–50]. Herein, we report our results in the establishment of a complementary one-pot domino approach without prior DG installation for the synthesis of diverse arylated alkylamides.

## Results and Discussion

To start the exploration, the reaction of AQ **1**, butyryl chloride (**2a**) and iodobenzene (**3a**) was selected as a model reaction. The primary reaction in the presence of Pd(OAc)_2_ provided smoothly the target product **4a** (entry 1, Table 1). The variation in the effect of catalyst loading proved that 5 mol % was proper (entries 2 and 3, Table 1). Further investigations using different palladium catalysts such as PdCl_2_, Pd/C, Pd(PPh_3_)_4_ did not show better results (entries 4–6, Table 1). The screening on the effect of base and solvent species demonstrated that K_2_CO_3_ and *p*-xylene were the best base additive and reaction medium, respectively (entries 7–15, Table 1). On the other hand, final optimization on the reaction temperature indicated that 120 °C was the favorable temperature (entries 16 and 17, Table 1).

**Table 1:** Optimization of reaction conditions.^a^

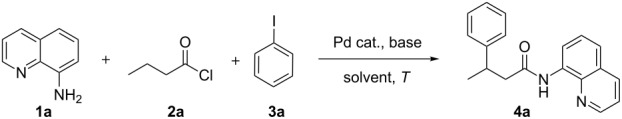				

Entry	Catalyst	Base	Solvent	Yield (%)^b^

1	Pd(OAc)_2_	K_2_CO_3_	*p*-xylene	75
2^c^	Pd(OAc)_2_	K_2_CO_3_	*p*-xylene	74
3^d^	Pd(OAc)_2_	K_2_CO_3_	*p*-xylene	51
4	PdCl_2_	K_2_CO_3_	*p*-xylene	52
5	Pd(PPh_3_)_4_	K_2_CO_3_	*p*-xylene	44
6	Pd/C	K_2_CO_3_	*p*-xylene	trace
7	Pd(OAc)_2_	Na_2_CO_3_	*p*-xylene	trace
8	Pd(OAc)_2_	NaHCO_3_	*p*-xylene	trace
9	Pd(OAc)_2_	Cs_2_CO_3_	*p*-xylene	63
10	Pd(OAc)_2_	KOH	*p*-xylene	trace
11	Pd(OAc)_2_	K_2_CO_3_	1,4-dioxane	26
12	Pd(OAc)_2_	K_2_CO_3_	CH_3_CN	trace
13	Pd(OAc)_2_	K_2_CO_3_	toluene	43
14	Pd(OAc)_2_	K_2_CO_3_	DMF	trace
15	Pd(OAc)_2_	K_2_CO_3_	DMSO	trace
16^e^	Pd(OAc)_2_	K_2_CO_3_	*p*-xylene	72
17^f^	Pd(OAc)_2_	K_2_CO_3_	*p*-xylene	55

^a^General conditions: **1a** (0.2 mmol), **2a** (0.2 mmol), **3a** (0.3 mmol), catalyst (5 mol %), base (0.4 mmol), solvent (2 mL), stirred at 120 °C or reflux (for solvents with lower bp) for 12 h. ^b^Isolated yield. ^c^Pd(OAc)_2_ (10 mol %). ^d^Pd(OAc)_2_ (3 mol %). ^e^The temperature was 130 °C. ^f^The temperature was 110 °C.

Following the above optimization experiments, the scope of this one-pot approach in the synthesis of arylated amides was then examined by subjecting different aryl iodides and acyl chlorides. According to the acquired results (Table 2), the acyl chlorides bearing chains of different length such as butanoyl, hexanoyl, octanoyl and dodecanoyl all exhibited good tolerance to the synthesis of the corresponding β-arylated products. Similarly, good compatibility was also observed in the component of aryl iodides. Functional groups of different features including alkyl, alkoxyl, halogen, nitro etc. were all well tolerated in the reaction, and the products were generally provided with good to excellent yields regardless of the different property of the substituent. A notable point was that the selective formation of single arylated products was kept even though diiodobenzenes were employed (**4f** and **4s**, Table 2). When 2-iodopyridine was employed, the expected reaction was not observed (entry 25, Table 2). Additionally, the reaction of 3-methylbutanoyl chloride, a secondary alkyl chloride, with iodobenzene was also found not practical, indicating the evident effect of steric hindrance to the reaction. Finally, the entry employing bromobenzene as reaction partner of **1** and **2a** provided only trace amounts of target product **4a**.

**Table 2:** Scope of the single β-arylation of alkylamides.^a^

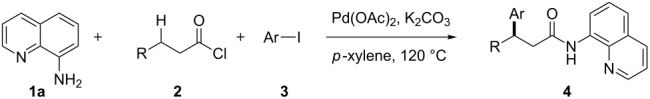				

Entry	R	Ar	Product	Yield (%)^b^

1	CH_3_	Ph	**4a**	72
2	CH_3_	4-MeC_6_H_4_	**4b**	65
3	CH_3_	4-OMeC_6_H_4_	**4c**	77
4	CH_3_	4-ClC_6_H_4_	**4d**	76
5	CH_3_	4-BrC_6_H_4_	**4e**	75
6	CH_3_	4-IC_6_H_4_	**4f**	78
7	CH_3_	4-NO_2_C_6_H_4_	**4g**	86
8	CH_3_	4-COMeC_6_H_4_	**4h**	72
9	*n*-C_3_H_7_	Ph	**4i**	74
10	*n*-C_3_H_7_	4-MeC_6_H_4_	**4j**	80
11	*n*-C_3_H_7_	4-OMeC_6_H_4_	**4k**	84
12	*n*-C_3_H_7_	4-ClC_6_H_4_	**4l**	85
13	*n*-C_3_H_7_	4-NO_2_C_6_H_4_	**4m**	76
14	*n*-C_3_H_7_	3-MeC_6_H_4_	**4n**	80
15	CH_3_(CH_2_)_4_	Ph	**4o**	78
16	CH_3_(CH_2_)_4_	4-MeC_6_H_4_	**4p**	65
17	CH_3_(CH_2_)_4_	4-OMeC_6_H_4_	**4q**	82
18	CH_3_(CH_2_)_4_	4-BrC_6_H_4_	**4r**	79
19	CH_3_(CH_2_)_4_	3-IC_6_H_4_	**4s**	67
20	CH_3_(CH_2_)_8_	Ph	**4t**	85
21	CH_3_(CH_2_)_8_	4-OMeC_6_H_4_	**4u**	78
22	CH_3_(CH_2_)_8_	4-ClC_6_H_4_	**4v**	75
23	CH_3_(CH_2_)_8_	4-BrC_6_H_4_	**4w**	70
24	CH_3_(CH_2_)_8_	4-NO_2_C_6_H_4_	**4x**	71
25	CH_3_	pyridine-2-yl	**–**	–

^a^General conditions: **1a** (0.3 mmol), **2** (0.3 mmol), **3** (0.45 mmol), Pd(OAc)_2_ (5 mol %), K_2_CO_3_ (0.6 mmol), *p*-xylene (2 mL), stirred at 120 °C for 12 h. ^b^Isolated yield.

**Scheme 1:**
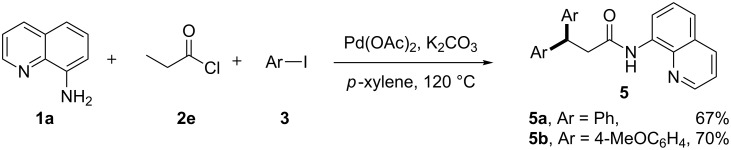
Double C–H arylation of *N*-AQ acetamide.

Interestingly, when less steric propionyl chloride **2e** was employed in the reactions with AQ and aryl iodides, the in situ generated propionamide intermedidates underwent selectively double β-C–H arylation to provide the corresponding 3,3-diarylamides **5** (Scheme 1).

**Scheme 2:**
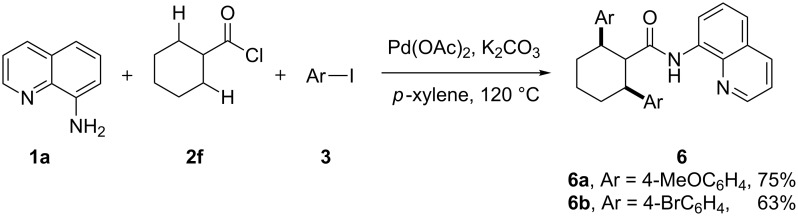
Double C–H arylation of *N*-AQ cyclohexylformamide.

What’s more, when cyclohexylformic acid (**2f**) was subjected with AQ and aryl iodides, the C–H bonds at the two identical β-carbon atoms were simultaneously arylated to yield 2,6-diarylcyclohexylformamides **6** (Scheme 2). The results in the production of all these single and double arylated products indicated the broad application scope of the present one-pot C–H arylation approach in the synthesis of diverse alkylamides.

## Conclusion

In conclusion, by employing directly 8-aminoquinoline, aliphatic acyl chlorides and aryl iodides as starting materials, the palladium-catalyzed, one-pot single or double C(sp^3^)-H β-arylation of the in situ generated alkylamides have been achieved. The distinct advantage of the present work lies in the considerably simplified operation process without requiring additional DG installation. The high efficiency, general application scope as well as the easy availability of starting materials enable this method as a practical complement to the present known strategies towards C–H activation-based arylated alkylamide synthesis.

## Supporting Information

File 1 Experimental details on the synthesis of all products **4**, **5** and **6**; full characterization data as well as ^1^H/^13^C NMR spectra of all products.
