# Supplementary material for: Synthesis of β-arylated alkylamides via Pd-catalyzed one-pot installation of a directing group and C(sp3)–H arylation
Source: Beilstein J Org Chem. 2016 Jun 3;12:1122–6. doi: 10.3762/bjoc.12.108 (PMC4902048; doi:10.3762/bjoc.12.108)

Synthesis of β-arylated alkylamides via Pd-catalyzed one-pot installation of a directing group and C(sp^3)–H arylation

Yunyun Liu*[1], Yi Zhang[1], Xiaoji Cao[2] and Jie-Ping Wan[1]

Address: [1]College of Chemistry and Chemical Engineering, Jiangxi Normal University, Nanchang 330022 P. R. China and [2]Research Center of Analysis and Measurement, Zhejiang University of Technology, 18 Chaowang Road, Hangzhou, Zhejiang 310014, P.R. China

Email: Yunyun Liu - chemliuyunyun@jxnu.edu.cn

*Corresponding author

Experimental details on the synthesis of all products 4, 5 and 6; full characterization data as well as ^{1}H/^{13}C NMR spectra of all products.

General information

All chemicals and solvents used in the experiments were obtained from J & K or Energy Chemical Co., Ltd. and were used directly without further treatment. ^{1}H and ^{13}C NMR spectra were recorded with a Bruker Avance 400 apparatus using $CDCl_3$ as solvent. The frequencies for ^{1}H NMR and ^{13}C NMR measurements were 400 MHz and 100 MHz, respectively. The chemical shifts were reported in ppm using TMS as internal standard. Melting points were measured with a X-4A instrument without correcting the temperature. HRMS data were obtained with a micrOTOF-QII Q-TOF mass spectrometer under ESI mode.

Synthesis of products 4, 5 and 6, general procedure

In a 25 mL round bottom flask were located 8-aminoquinoline (**1**, 0.3 mmol), acyl chloride **2** (0.3 mmol), iodobenzene **3** (0.45 mmol)/(0.9 mmol in the synthesis of **5** and **6**), $Pd(OAc)_2$ (0.015 mmol), K_2CO_3 (0.6 mmol) and *p*-xylene (2 mL). The mixture was stirred at 120 °C for 12 h. Upon completion, the reaction was allowed to cool down to room temperature, and 10 mL water was added. The resulting heterogeneous mixture was extracted with ethyl acetate (3 × 10 mL). The combined organic phase was dried over anhydrous Na_2SO_4. After filtering, the acquired solution was subjected to reduced pressure to remove the solvent. The residue was then purified by with silica gel column chromatography to give pure products by using mixed petroleum ether and ethyl acetate (V_{PET}/V_{EA} = 10:1).

Characterization data of all products

3-Phenyl-*N*-(quinolin-8-yl)butanamide (**4a**).[1] Yield: 63 mg, 72%; colorless oil; ^{1}H NMR (400 MHz, $CDCl_3$): δ = 9.65 (s, 1 H), 8.68-8.66 (m, 2 H), 8.03-8.01 (d, *J* = 8.4 Hz, 1 H), 7.43-7.36 (m, 2 H), 7.31 (dd, *J* = 8.0, 4.0 Hz, 1 H), 7.25-7.19 (m, 4 H), 7.11-7.08 (m, 1 H), 3.45-3.37 (m, 1 H), 2.80 (dd, *J* = 14.4, 6.4 Hz, 1 H), 2.67 (dd, *J* = 14.4, 8.0 Hz, 1 H), 1.32 (d, *J* = 6.4 Hz, 3 H); ^{13}C NMR (100 MHz, $CDCl_3$): δ = 169.3, 147.0, 144.9, 137.2, 135.3, 133.3, 127.5, 126.8, 126.3, 125.8, 125.3, 120.5, 120.4, 115.4, 45.8, 35.8, 20.8.

***N*-(Quinolin-8-yl)-3-(*p*-tolyl)butanamide** (**4b**).[1] Yield: 59 mg, 65%; colorless oil; ^{1}H NMR (400 MHz, $CDCl_3$): δ = 9.75 (s, 1 H), 8.78-8.77 (m, 2 H), 8.14-8.12 (d, *J* = 8.0 Hz, 1 H), 7.53-7.46 (m, 2 H), 7.43 (dd, *J* = 8.4, 4.4 Hz, 1H), 7.23 (d, *J* = 8.0 Hz, 2 H), 7.12 (d, *J* = 7.2 Hz, 2 H), 3.50-3.43 (m, 1 H), 2.88 (dd, *J* = 14.0, 6.4 Hz, 1H), 2.76 (dd, *J* = 14.0, 8.0 Hz, 1H), 2.30 (s, 3 H), 1.40 (d, *J* = 6.8 Hz, 3 H); ^{13}C NMR (100 MHz, $CDCl_3$): δ = 170.5, 148.0, 143.0, 138.2, 136.4, 135.9, 134.4, 129.3, 127.9, 127.4, 126.7, 121.6, 121.4, 116.5, 47.0, 36.5, 22.0, 21.0.

3-(4-Methoxyphenyl)-*N*-(quinolin-8-yl)butanamide (**4c**).[1] Yield: 74 mg, 77%; colorless oil; ^{1}H NMR (400 MHz, $CDCl_3$): δ = 9.72 (s, 1 H), 8.77-8.75 (m, 2 H), 8.12 (d, *J* = 8.0 Hz, 1 H), 7.53-7.45 (m, 2 H), 7.42 (dd, *J* = 8.0, 4.4 Hz, 1 H), 7.24 (d, *J* = 8.0 Hz, 2 H), 6.84 (d, *J* = 8.8 Hz, 2 H), 3.74 (s, 3 H), 3.49-3.41 (m, 1 H), 2.84 (dd, *J* = 14.4, 7.2 Hz, 1 H), 2.74 (dd, *J* = 14.4, 8.4 Hz, 1 H), 1.38 (d, *J* = 7.2 Hz, 3 H); ^{13}C NMR (100 MHz, $CDCl_3$): δ = 170.5, 158.1, 148.0, 138.2, 138.0, 136.4, 134.4, 127.9, 127.8, 127.4, 121.5, 121.4, 116.5, 114.0, 55.2, 47.2, 36.1, 22.1.

3-(4-Chlorophenyl)-*N*-(quinolin-8-yl)butanamide (**4d**).[1] Yield: 74 mg, 76%; colorless oil; ^{1}H NMR (400 MHz, $CDCl_3$): δ = 9.71 (s, 1 H), 8.77-8.73 (m, 2 H), 8.14 (dd, *J* = 8.4, 1.6 Hz, 1 H), 7.54-7.48 (m, 2 H), 7.45 (dd, *J* = 8.4, 4.4 Hz, 1 H), 7.26 (s, 4 H), 3.52-3.43 (m, 1 H), 2.87-2.74 (m, 2 H), 1.38 (d, *J* = 6.4 Hz, 3 H); ^{13}C NMR (100 MHz, $CDCl_3$): δ = 170.0, 148.0, 144.4, 138.2, 136.4, 134.3, 132.0, 128.7, 128.3, 127.9, 127.4, 121.6, 121.5, 116.6, 46.7, 36.3, 21.8.

3-(4-Bromophenyl)-*N*-(quinolin-8-yl)butanamide (**4e**).[1] Yield: 83 mg, 75%; colorless oil; ^{1}H NMR (400 MHz, $CDCl_3$): δ = 9.70 (s, 1 H), 8.77-8.73 (m, 2 H), 8.15 (d, *J* = 8.0 Hz, 1 H), 7.53-7.47 (m, 2 H), 7.46-7.40 (m, 3 H), 7.20 (d, *J* = 8.4 Hz, 2 H), 3.49-3.44 (m, 1 H), 2.87-2.74 (m, 2 H), 1.38 (d, *J* = 6.8 Hz, 3 H); ^{13}C NMR (100 MHz, $CDCl_3$): δ = 169.9, 148.0, 144.9, 138.1, 136.5, 134.2, 131.7, 128.7, 127.9, 127.4, 121.6, 121.5, 120.1, 116.6, 46.6, 36.4, 21.8.

3-(4-Iodophenyl)-*N*-(quinolin-8-yl)butanamide (**4f**).[1] Yield: 97 mg, 78%; colorless oil; ^{1}H NMR (400 MHz, $CDCl_3$): δ = 9.70 (s, 1 H), 8.76-8.72 (m, 2 H), 8.11 (dd, *J* = 8.4, 1.2 Hz, 1 H), 7.59 (d, *J* = 8.4 Hz, 2 H), 7.51-7.45 (m, 2 H), 7.41 (dd, *J* = 8.0, 4.0 Hz, 1 H), 7.06 (d, *J* = 8.4 Hz, 2 H), 3.48-3.39 (m, 1 H), 2.85-2.71 (m, 2 H), 1.36 (d, *J* = 8.0 Hz, 3 H); ^{13}C NMR (100 MHz, $CDCl_3$): δ = 169.9, 148.1, 145.6, 138.1, 137.6, 136.4, 134.3, 129.0, 127.9, 127.4, 121.6, 116.6, 91.6, 46.6, 36.5, 21.8.

3-(4-Nitrophenyl)-*N*-(quinolin-8-yl)butanamide (**4g**).[2] Yield: 86 mg, 86%; yellow solid, mp 138-140°C; ^{1}H NMR (400 MHz, $CDCl_3$): δ = 9.72 (s, 1 H), 8.75-8.69 (m, 2 H), 8.13 (d, *J* = 8.8 Hz, 3 H), 7.52-7.42 (m, 5 H), 3.67-3.58 (m, 1 H), 2.92-2.82 (m, 2 H), 1.43 (d, *J* = 6.8 Hz, 3 H); ^{13}C NMR (100 MHz, $CDCl_3$): δ = 169.3, 153.6, 148.1,

146.6, 138.1, 136.5, 134.1, 127.9, 127.8, 127.3, 123.9, 121.7, 121.7, 116.6, 46.1, 36.7, 21.6.

3-(4-Acetylphenyl)-*N*-(quinolin-8-yl)butanamide (**4h**). Yield: 72 mg, 72%; yellow oil; ^{1}H NMR (400 MHz, $CDCl_3$): δ = 9.73 (s, 1 H), 8.75-8.73 (m, 2 H), 8.12 (d, *J* = 8.0 Hz, 1 H), 7.89 (d, *J* = 8.4 Hz, 2 H), 7.52-7.46 (m, 2 H), 7.43-7.40 (m, 3 H), 3.60-3.54 (m, 1 H), 2.91-2.78 (m, 2 H), 2.54 (s, 3 H), 1.42 (d, *J* = 6.8 Hz, 3 H);^{13}C NMR (100 MHz, $CDCl_3$): δ = 197.8, 169.8, 151.6, 148.1, 138.1, 136.4, 135.5, 134.2, 128.8, 128.7, 127.9, 127.4, 127.1, 121.6, 116.5, 46.3, 36.8, 26.6, 21.6; HRMS (ESI): m/z $[M + H]^+$ calcd for $C_{21}H_{21}N_2O_2$: 333.1598; found: 333.1593.

3-Phenyl-*N*-(quinolin-8-yl)hexanamide (**4i**).[1] Yield: 71 mg, 74%; colorless oil; ^{1}H NMR (400 MHz, $CDCl_3$): δ = 9.69 (s, 1 H), 8.74-8.72 (m, 2 H), 8.10 (d, *J* = 8.0 Hz, 1 H), 7.50-7.43 (m, 2 H), 7.40 (dd, *J* = 8.0, 4.0 Hz, 1 H), 7.28 (d, *J* = 3.6 Hz, 4 H), 7.18-7.14 (m, 1 H), 3.35-3.28 (m, 1 H), 2.83 (d, *J* = 7.2 Hz, 2 H), 1.80-1.65 (m, 2 H), 1.28-1.17 (m, 2 H), 0.86 (t, *J* = 7.4 Hz, 3 H); ^{13}C NMR (100 MHz, $CDCl_3$): δ = 170.5, 148.0, 144.4, 138.2, 136.4, 134.4, 128.5, 127.9, 127.6, 127.4, 126.4, 121.5, 121.4, 116.5, 45.8, 42.4, 38.4, 20.6, 14.0.

***N*-(Quinolin-8-yl)-3-(*p*-tolyl)hexanamide** (**4j**). Yield: 80 mg, 80%; colorless oil; ^{1}H NMR (400 MHz, $CDCl_3$): δ = 9.69 (s, 1 H), 8.74 (s, 2 H), 8.10 (d, *J* = 8.0 Hz, 1H), 7.50-7.43 (m, 2 H), 7.40 (dd, *J* = 8.0, 4.0 Hz, 1 H), 7.17 (d, *J* = 8.0 Hz, 2 H), 7.08 (d, *J* = 7.6 Hz, 2 H), 3.31-2.24 (m, 1 H), 2.82 (d, *J* = 7.2 Hz, 2 H), 2.27 (s, 3 H), 1.78-1.63 (m, 2 H), 1.25-1.20 (m, 2 H), 0.85 (t, *J* = 7.0 Hz, 3 H); ^{13}C NMR (100 MHz, $CDCl_3$): δ = 170.6, 147.9, 141.3, 138.2, 136.3, 135.8, 134.5, 129.2, 127.9, 127.4, 121.5, 121.3, 116.5, 46.0, 42.0, 38.5, 21.0, 20.6, 14.0; HRMS (ESI): m/z $[M + H]^+$ calcd for $C_{22}H_{25}N_2O$: 333.1961; found: 333.1946.

3-(4-Methoxyphenyl)-*N*-(quinolin-8-yl)hexanamide (**4k**). Yield: 88 mg, 84%; colorless oil; ^{1}H NMR (400 MHz, $CDCl_3$): δ = 9.66 (s, 1 H), 8.73 (s, 2 H), 8.10 (d, *J* = 8.0 Hz, 1 H), 7.50-7.43 (m, 2 H), 7.40 (dd, *J* = 8.0, 4.0 Hz, 1 H), 7.20 (d, *J* = 8.4 Hz, 2 H), 6.81 (d, *J* = 8.0 Hz, 2 H), 3.72 (s, 3 H), 3.26 (s, 1 H), 2.82-2.79 (m, 2 H), 1.75-1.63 (m, 2 H), 1.25-1.21 (m, 2 H), 0.86 (t, *J* = 7.0 Hz, 3 H); ^{13}C NMR (100

MHz, $CDCl_3$): δ = 170.6, 158.1, 147.9, 138.2, 136.4, 136.3, 134.4, 128.5, 127.9, 127.4, 121.5, 121.4, 116.5, 113.9, 55.1, 46.1, 41.6, 38.6, 20.6, 14.0; HRMS (ESI): m/z $[M + H]^+$ calcd for $C_{22}H_{25}N_2O_2$: 349.1911; found: 349.1924.

3-(4-Chlorophenyl)-*N*-(quinolin-8-yl)hexanamide (**4l**). Yield: 90 mg, 85%; colorless oil; ^1H NMR (400 MHz, $CDCl_3$): δ = 9.65 (s, 1 H), 8.74-8.70 (m,2 H), 8.10 (d, *J* = 8.0 Hz, 1 H), 7.50-7.44 (m, 2 H), 7.40 (dd, *J* = 8.0, 4.0 Hz, 1 H), 7.22 (d, *J* = 3.6 Hz, 4 H), 3.29 (s, 1 H), 2.86-2.74 (m, 2 H), 1.75-1.62 (m, 2 H), 1.24-1.18 (m, 2 H), 0.86 (t, *J* = 7.0 Hz, 3 H); ^{13}C NMR (100 MHz, $CDCl_3$): δ = 170.1, 148.0, 142.8, 138.2, 136.4, 134.3, 132.0, 129.0, 128.7, 127.9, 127.4, 121.6, 121.5, 116.5, 45.7, 41.8, 38.4, 20.5, 14.0; HRMS (ESI): m/z $[M + H]^+$ calcd for $C_{21}H_{22}ClN_2O$: 353.1415; found: 353.1427.

3-(4-Nitrophenyl)-*N*-(quinolin-8-yl)hexanamide (**4m**). Yield: 83 mg, 76%; yellow oil; ^1H NMR (400 MHz, $CDCl_3$): δ = 9.68 (s, 1 H), 8.74-8.66 (m, 2 H), 8.14-8.12 (m, 3 H), 7.49-7.41 (m, 5 H), 3.50-3.43 (m, 1 H), 2.96-2.80 (m, 2 H), 1.81-1.67 (m, 2 H), 1.28-1.15 (m, 2 H), 0.88 (t, *J* = 7.2 Hz, 3 H); ^{13}C NMR (100 MHz, $CDCl_3$): δ = 169.4, 152.4, 148.1, 146.6, 138.1, 136.5, 134.1, 128.5, 127.9, 127.3, 123.8, 121.7, 121.7, 116.5, 45.0, 42.2, 38.1, 20.5, 13.9; HRMS (ESI): m/z $[M + H]^+$ calcd for $C_{21}H_{22}N_3O_3$: 364.1656; found: 364.1661.

***N*-(Quinolin-8-yl)-3-(*m*-tolyl)hexanamide** (**4n**). Yield: 80 mg, 80%; colorless oil; ^1H NMR (400 MHz, $CDCl_3$): δ = 9.68 (s, 1 H), 8.74-8.73 (m, 2 H), 8.09 (d, *J* = 8.4 Hz, 1 H), 7.50-7.42 (m, 2 H), 7.38 (dd, *J* = 8.0, 4.0 Hz, 1 H), 7.16 (t, *J* = 7.4 Hz, 1 H), 7.08 (d, *J* = 10.0 Hz, 2 H), 6.96 (d, *J* = 7.2 Hz, 1 H), 3.31-3.23 (m, 1 H), 2.82 (d, *J* = 7.2 Hz, 2 H), 2.29 (s, 3 H), 1.78-1.64 (m, 2 H), 1.28-1.19 (m, 2 H), 0.86 (t, *J* = 7.0 Hz, 3 H); ^{13}C NMR (100 MHz, $CDCl_3$): δ = 170.6, 148.0, 144.3, 138.2, 138.0, 136.3, 134.5, 128.4, 128.3, 127.9, 127.4, 127.2, 124.6, 121.5, 121.4, 116.5, 45.9, 42.4, 38.5, 21.5, 20.6, 14.1; HRMS (ESI): m/z $[M + H]^+$ calcd for $C_{22}H_{25}N_2O$: 333.1961; found: 333.1946.

3-Phenyl-*N*-(quinolin-8-yl)octanamide (**4o**). Yield: 81 mg, 78%; colorless oil; ^1H NMR (400 MHz, $CDCl_3$): δ = 9.68 (s, 1 H), 8.75-8.72 (m, 2 H), 8.11 (dd, *J* = 8.0, 1.2

Hz, 1 H), 7.51-7.44 (m, 2 H), 7.41 (dd, *J* = 8.0, 4.0 Hz, 1 H), 7.29 (d, *J* = 3.6 Hz, 4 H), 7.18-7.16 (m, 1 H), 3.33-3.26 (m, 1 H), 2.85-2.83 (m, 2 H), 1.79-1.67 (m, 2 H), 1.27-1.23 (m, 6 H), 0.81 (s, 3 H); ^{13}C NMR (100 MHz, $CDCl_3$): δ = 170.5, 148.0, 144.4, 138.2, 136.4, 134.4, 128.5, 127.9, 127.6, 127.4, 126.4, 121.5, 121.4, 116.5, 45.9, 42.6, 36.2, 31.8, 27.1, 22.5, 14.0; HRMS (ESI): m/z $[M + H]^+$ calcd for $C_{23}H_{27}N_2O$: 347.2118; found: 347.2119.

N-(Quinolin-8-yl)-3-(p-tolyl)octanamide (**4p**). Yield: 67 mg, 65%; colorless oil; ^{1}H NMR (400 MHz, $CDCl_3$): δ = 9.68 (s, 1 H), 8.75-8.73 (m, 2 H), 8.12 (d, *J* = 8.4 Hz, 1 H), 7.51-7.44 (m, 2 H), 7.41 (dd, *J* = 8.4, 4.4 Hz, 1 H), 7.17 (d, *J* = 8.0 Hz, 2 H), 7.08 (d, *J* = 7.6 Hz, 2 H), 3.29-3.22 (m, 1 H), 2.83-2.80 (m, 2 H), 2.27 (s, 3 H), 1.77-1.64 (m, 2 H), 1.22 (s, 6 H), 0.81 (s, 3 H); ^{13}C NMR (100 MHz, $CDCl_3$): δ = 170.6, 147.9, 141.3, 138.2, 136.4, 135.8, 134.5, 129.2, 129.1, 127.9, 127.4, 121.5, 121.3, 116.5, 46.0, 42.2, 36.3, 31.8, 27.1, 22.5, 21.0, 14.1; HRMS (ESI): m/z $[M + H]^+$ calcd for $C_{24}H_{29}N_2O$: 361.2274; found: 361.2258.

3-(4-Methoxyphenyl)-*N*-(quinolin-8-yl)octanamide (**4q**). Yield: 92 mg, 82%; colorless oil; ^{1}H NMR (400 MHz, $CDCl_3$): δ = 9.65 (s, 1 H), 8.73-8.71 (m, 2 H), 8.07 (d, *J* = 8.4 Hz, 1 H), 7.48-7.41 (m, 2 H), 7.37 (dd, *J* = 8.0, 4.0 Hz, 1 H), 7.19 (d, *J* = 8.4 Hz, 2 H), 6.81 (d, *J* = 8.4 Hz, 2 H), 3.71 (s, 3 H), 3.27-3.20 (m, 1 H), 2.85-2.74 (m, 2 H), 1.76-1.63 (m, 2 H), 1.22 (s, 6 H), 0.81 (s, 3 H); ^{13}C NMR (100 MHz, $CDCl_3$): δ = 170.6, 158.1, 148.0, 138.2, 136.4, 136.3, 134.4, 128.4, 127.9, 127.4, 121.5, 121.4, 116.4, 113.9, 55.1, 46.1, 41.9, 36.4, 31.8, 27.1, 22.6, 14.1; HRMS (ESI): m/z $[M + H]^+$ calcd for $C_{24}H_{29}N_2O_2$: 377.2224; found: 377.2214.

3-(4-Bromophenyl)-*N*-(quinolin-8-yl)octanamide (**4r**). Yield: 100 mg, 79%; colorless oil; ^{1}H NMR (400 MHz, $CDCl_3$): δ = 9.64 (s, 1 H), 8.76-8.69 (m, 2 H), 8.14-8.12 (m, 1 H), 7.51-7.38 (m, 5 H), 7.16 (d, *J* = 8.4 Hz, 2 H), 3.30-3.22 (m, 1 H), 2.87-2.74 (m, 2 H), 1.78-1.62 (m, 2 H), 1.22 (s, 6 H), 0.82 (s, 3 H); ^{13}C NMR (100 MHz, $CDCl_3$): δ = 170.0, 148.0, 143.4, 138.1, 136.4, 134.3, 131.6, 129.4, 127.9, 127.4, 121.6, 121.5, 120.1, 116.5, 45.7, 42.1, 36.1, 31.7, 27.0, 22.5, 14.1; HRMS (ESI): m/z $[M + H]^+$ calcd for $C_{23}H_{26}BrN_2O$: 425.1223; found: 425.1212.

3-(3-Iodophenyl)-*N*-(quinolin-8-yl)octanamide (**4s**). Yield: 95 mg, 67%; colorless oil; ^{1}H NMR (400 MHz, $CDCl_3$): δ = 9.68 (s, 1 H), 8.78 (d, *J* = 4.4 Hz, 1 H), 8.71 (d, *J* = 6.8 Hz, 1 H), 8.14 (d, *J* = 8.4 Hz, 1 H), 7.65 (s, 1 H), 7.52-7.42 (m, 4 H), 7.24 (d, *J* = 8.8 Hz, 1 H), 6.99 (t, *J* = 7.8 Hz, 1 H), 3.26-3.19 (m, 1 H), 2.87-2.76 (m, 2 H), 1.78-1.63 (m, 2 H), 1.23 (s, 6 H), 0.82 (s, 3 H); ^{13}C NMR (100 MHz, $CDCl_3$): δ = 170.0, 148.0, 147.0, 138.0, 136.5, 136.4, 135.5, 134.2, 130.3, 127.9, 127.4, 127.1, 121.5, 121.5, 116.7, 94.8, 45.5, 42.3, 36.1, 31.7, 27.0, 22.5, 14.0; HRMS (ESI):m/z $[M + H]^+$ calcd for $C_{23}H_{26}IN_2O$: 473.1084; found: 473.1084.

3-Phenyl-*N*-(quinolin-8-yl)dodecanamide (**4t**).[1] Yield: 102 mg, 85%; colorless oil; ^{1}H NMR (400 MHz, $CDCl_3$): δ = 9.68 (s, 1 H), 8.73 (d, *J* = 8.0 Hz, 2 H), 8.06 (d, *J* = 7.6 Hz, 1 H), 7.48-7.40 (m, 2 H), 7.36 (dd, *J* = 8.0, 4.0 Hz, 1 H), 7.28 (s, 4 H), 7.17-7.15 (m, 1 H), 3.31-3.26 (m, 1 H), 2.87-2.78 (m, 2 H), 1.79-1.67 (m, 2 H), 1.18 (s, 14 H), 0.85 (t, *J* = 6.6 Hz, 3 H); ^{13}C NMR (100 MHz, $CDCl_3$): δ = 170.4, 148.0, 144.5, 138.2, 136.3, 134.4, 128.5, 127.9, 127.6, 127.4, 126.4, 121.5, 121.4, 116.4, 45.9, 42.6, 36.3, 31.9, 29.6, 29.6, 29.5, 29.3, 27.5, 22.7, 14.2.

3-(4-Methoxyphenyl)-*N*-(quinolin-8-yl)dodecanamide (**4u**). Yield: 101 mg, 78%; colorless oil; ^{1}H NMR (400 MHz, $CDCl_3$): δ = 9.66 (s, 1 H), 8.74-8.72 (m, 2 H), 8.08 (d, *J* = 8.0 Hz, 1 H), 7.49-7.41 (m, 2 H), 7.38 (dd, *J* = 8.0, 4.0 Hz, 1 H), 7.20 (d, *J* = 8.8 Hz, 2 H), 6.81 (d, *J* = 8.4 Hz, 2 H), 3.71 (s, 3 H), 3.28-3.20 (m, 1 H), 2.85-2.74 (m, 2 H), 1.78-1.62 (m, 2 H), 1.19 (s, 14 H), 0.85 (t, *J* = 7.0 Hz, 3 H); ^{13}C NMR (100 MHz, $CDCl_3$): δ = 170.6, 158.1, 147.9, 138.2, 136.4, 136.4, 134.4, 128.4, 127.9, 127.4, 121.5, 121.3, 116.5, 113.9, 55.1, 46.2, 41.9, 36.4, 31.9, 29.6, 29.6, 29.3, 27.5, 22.7, 14.2; HRMS (ESI): m/z $[M + H]^+$ calcd for $C_{28}H_{37}N_2O_2$: 433.2850; found: 433.2862.

3-(4-Chlorophenyl)-*N*-(quinolin-8-yl)dodecanamide (**4v**). Yield: 98 mg, 75%; colorless oil; ^{1}H NMR (400 MHz, $CDCl_3$): δ = 9.64 (s, 1 H), 8.74-8.70 (m, 2 H), 8.10 (d, *J* = 7.2 Hz, 1 H), 7.50-7.44 (m, 2 H), 7.40 (dd, *J* = 8.0, 4.4 Hz, 1 H), 7.25-7.20 (m, 4H), 3.27 (s, 1 H), 2.86-2.74 (m, 2 H), 1.76-1.64 (m, 2 H), 1.19 (s, 14 H), 0.85 (t, *J* = 6.8 Hz, 3 H); ^{13}C NMR (100 MHz, $CDCl_3$): δ = 170.0, 148.0, 142.9, 138.2, 136.3,

134.3, 132.0, 129.0, 128.7, 127.9, 127.4, 121.6, 121.5, 116.5, 45.7, 42.1, 36.2, 31.9, 29.5, 29.5, 29.3, 27.4, 22.7, 14.1; HRMS (ESI): m/z $[M + H]^+$ calcd for $C_{27}H_{34}ClN_2O$: 437.2354; found: 437.2364.

3-(4-Bromophenyl)-*N*-(quinolin-8-yl)dodecanamide (**4w**). Yield: 112 mg, 70%; colorless oil; ^1H NMR (400 MHz, $CDCl_3$): δ = 9.65 (s, 1 H), 8.76-8.70 (m, 2 H), 8.14-8.12 (m, 1 H), 7.52-7.38 (m, 5 H), 7.16 (d, *J* = 8.4 Hz, 2 H), 3.30-3.22 (m, 1 H), 2.87-2.75 (m, 2 H), 1.79-1.63 (m, 2 H), 1.19 (s, 14 H), 0.86 (t, *J* = 6.8 Hz, 3 H); ^{13}C NMR (100 MHz, $CDCl_3$): δ = 170.0, 148.0, 143.4, 138.1, 136.4, 134.3, 131.6, 129.4, 127.9, 127.4, 121.6, 121.5, 120.1, 116.5, 45.7, 42.1, 36.2, 31.9, 29.5, 29.5, 29.5, 29.3, 27.4, 22.7, 14.1; HRMS (ESI): m/z $[M + H]^+$ calcd for $C_{27}H_{34}BrN_2O$: 481.1849; found: 481.1840.

3-(4-Nitrophenyl)-*N*-(quinolin-8-yl)dodecanamide (**4x**). Yield: 95 mg, 71%; yellow oil; ^1H NMR (400 MHz, $CDCl_3$): δ = 9.67 (s, 1 H), 8.74-8.66 (m, 2 H), 8.13 (d, *J* = 8.0 Hz, 3 H), 7.48-7.41 (m, 5 H), 3.46-3.41 (m, 1 H), 2.96-2.80 (m, 2 H), 1.86-1.63 (m, 2 H), 1.25-1.20 (m, 14 H), 0.85 (t, *J* = 6.8 Hz, 3 H); ^{13}C NMR (100 MHz, $CDCl_3$): δ = 169.4, 152.4, 148.0, 146.6, 138.1, 136.5, 134.0, 128.5, 127.9, 127.4, 123.8, 121.7, 121.6, 116.7, 45.1, 42.5, 36.0, 31.8, 29.6, 29.5, 29.4, 29.3, 27.3, 22.7, 14.1; HRMS (ESI): m/z $[M + H]^+$ calcd for $C_{27}H_{34}N_3O_3$: 448.2595; found: 448.2593.

3,3-Diphenyl-*N*-(quinolin-8-yl)propanamide (**5a**).[1] Yield: 71 mg, 67%; colorless oil; ^1H NMR (400 MHz, $CDCl_3$): δ = 9.77 (s, 1 H), 8.74-8.69 (m, 2 H), 8.10 (d, *J* = 8.0 Hz, 1 H), 7.49-7.45 (m, 2 H), 7.40 (dd, *J* = 8.0, 4.4 Hz, 1 H), 7.34 (d, *J* = 7.2 Hz, 4 H), 7.27 (t, *J* = 7.4 Hz, 4 H), 7.16 (t, *J* = 7.2 Hz, 2 H), 4.79 (t, *J* = 7.4 Hz, 1 H), 3.31 (d, *J* = 7.6 Hz, 2 H); ^{13}C NMR (100 MHz, $CDCl_3$): δ = 169.6, 148.0, 143.8, 138.1, 136.4, 134.3, 128.6, 127.8, 127.4, 126.5, 121.5, 121.5, 116.6, 47.2, 44.4.

3,3-Bis(4-methoxyphenyl)-*N*-(quinolin-8-yl)propanamide (**5b**).[2] Yield: 86 mg, 70%; colorless oil; ^1H NMR (400 MHz, $CDCl_3$): δ = 9.75 (s, 1 H), 8.74-8.69 (m, 2 H), 8.12 (d, *J* = 8.0 Hz, 1 H), 7.50-7.40 (m, 3 H), 7.23 (d, *J* = 8.4 Hz, 4 H), 6.80 (d, *J* = 8.4 Hz, 4 H), 4.67 (t, *J* = 7.6 Hz, 1 H), 3.72 (s, 6 H), 3.25 (d, *J* = 7.6 Hz, 2 H); ^{13}C

NMR (100 MHz, $CDCl_3$): δ = 169.9, 158.1, 147.9, 138.1, 136.5, 136.3, 134.3, 128.7, 127.9, 127.4, 121.5, 121.5, 116.7, 114.0, 55.2, 45.7, 44.9.

2,6-Bis(4-methoxyphenyl)-*N*-(quinolin-8-yl)cyclohexane-1-carboxamide (**6a**).[2] Yield: 100 mg, 75%; white solid, mp 185-187°C; ^{1}H NMR (400 MHz, $CDCl_3$): δ = 8.63 (s, 1 H), 8.51-8.43 (m, 2 H), 7.95 (d, *J* = 7.6 Hz, 1 H), 7.38 (t, *J* = 7.8 Hz, 1 H), 7.31 (d, *J* = 8.0 Hz, 1 H), 7.22 (d, *J* = 8.4 Hz, 5 H), 6.62 (d, *J* = 8.4 Hz, 4 H), 3.49 (s, 6 H), 3.11-3.08 (m, 3 H), 2.76-2.67 (m, 2 H), 2.22-2.19 (m, 1 H), 1.79-1.76 (m, 2 H), 1.65-1.61 (m, 1 H); ^{13}C NMR (100 MHz, $CDCl_3$): δ = 171.3, 157.9, 147.3, 137.9, 136.4, 135.8, 134.3, 128.4, 127.5, 127.1, 121.0, 120.9, 116.1, 113.6, 57.4, 55.0, 47.0, 26.7, 25.9.

2,6-Bis(4-bromophenyl)-N-(quinolin-8-yl)cyclohexane-1-carboxamide (**6b**). Yield: 106 mg, 63%; white solid, mp 230-232°C; ^{1}H NMR (400 MHz, $CDCl_3$): δ = 8.62 (s, 1 H), 8.50-8.49 (m, 1 H), 8.41-8.43 (m, 1 H), 8.02 (d, *J* = 7.6 Hz, 1 H), 7.43-7.37 (m, 2 H), 7.32 (dd, *J* = 8.0, 4.0 Hz, 1 H), 7.22-7.17 (m, 8 H), 3.12-3.08 (m, 3 H), 2.76-2.65 (m, 2 H), 2.23-2.19 (m, 1 H), 1.79-1.75 (m, 2 H), 1.64-1.61 (m, 1 H); ^{13}C NMR (100 MHz, $CDCl_3$): δ = 170.4, 166.6, 147.8, 142.9, 137.9, 135.9, 133.7, 131.3, 129.3, 127.6, 126.9, 121.4, 120.3, 116.2, 56.5, 47.2, 26.4, 25.4;HRMS (ESI):m/z $[M + H]^+$ calcd for $C_{28}H_{25}Br_2N_2O$: 563.0328; found: 563.0324.

References

1) Pan, F.; Shen, P.-X.; Zhang,L.-S.; Wang,X.; Shi, Z.-J. *Org. Lett*. **2013**, *15*, 4758.

2) Wei, Y.; Tang, H.; Cong, X.; Rao, B.; Wu, C.; Zeng, X. *Org. Lett.* **2014**, *16*, 2248.

^{1}H and ^{13}C NMR spectra of all products

^{1}H and ^{13}C NMR spectra of 4a

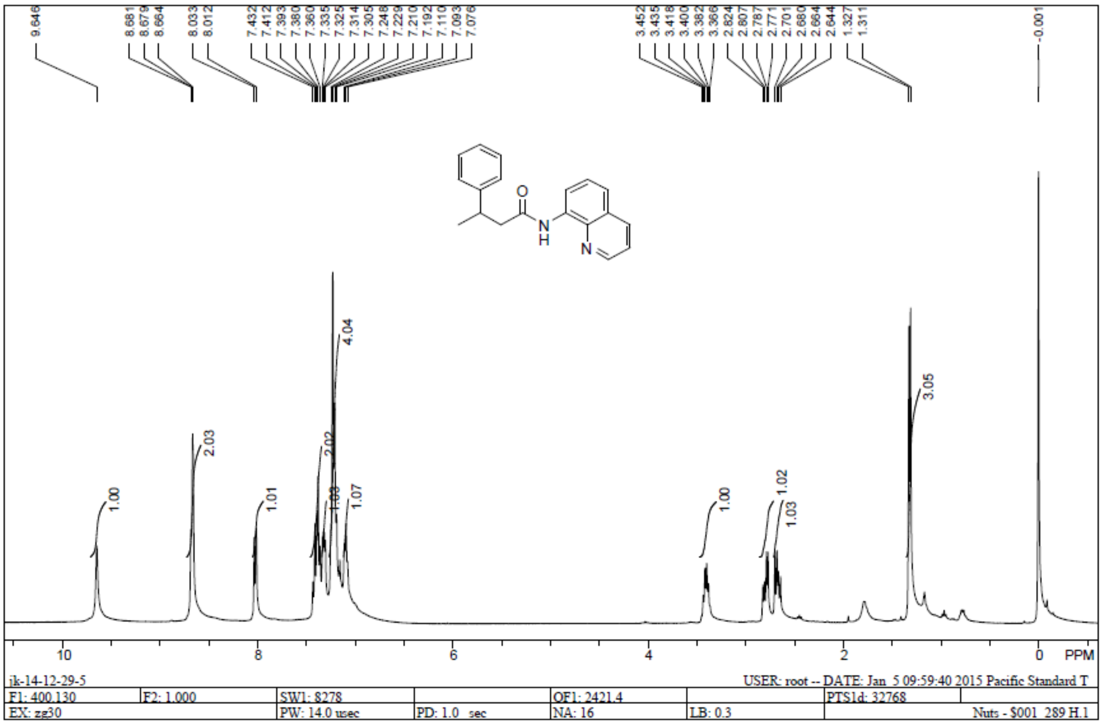

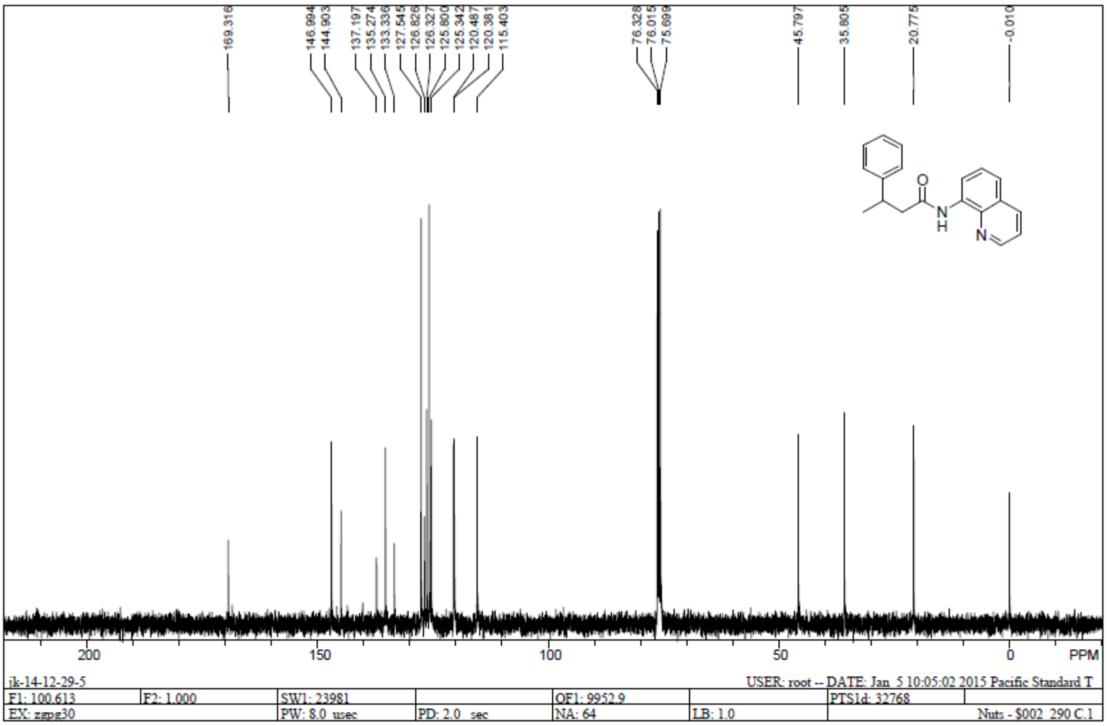

^{1}H and ^{13}C NMR spectra of 4b

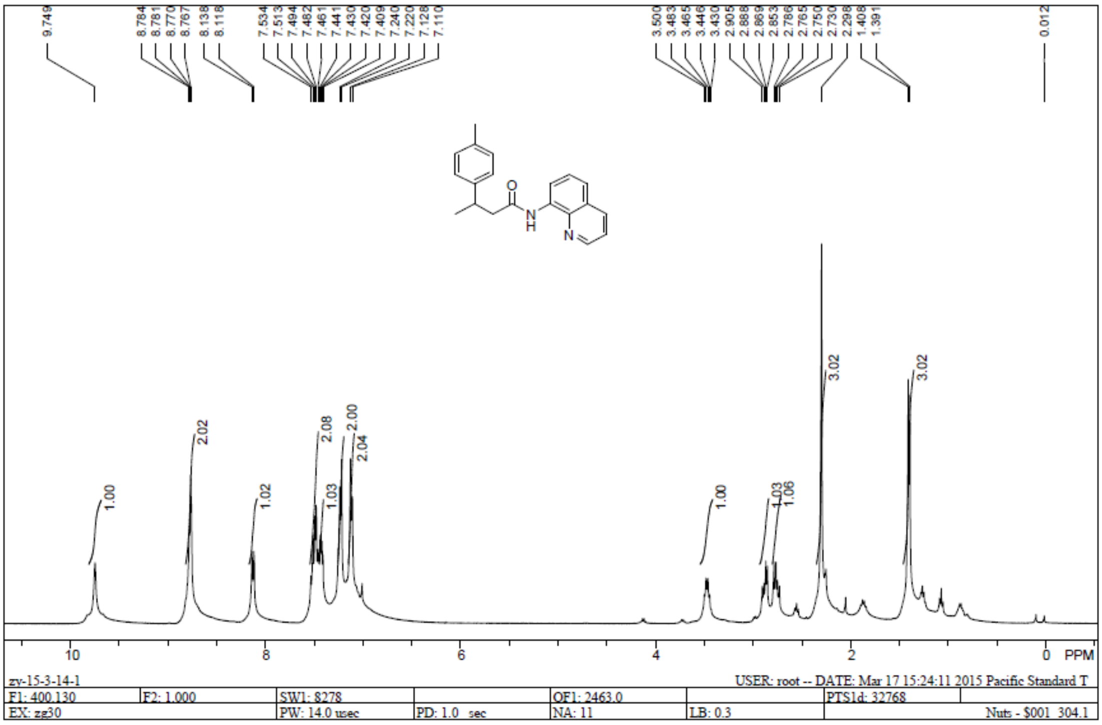

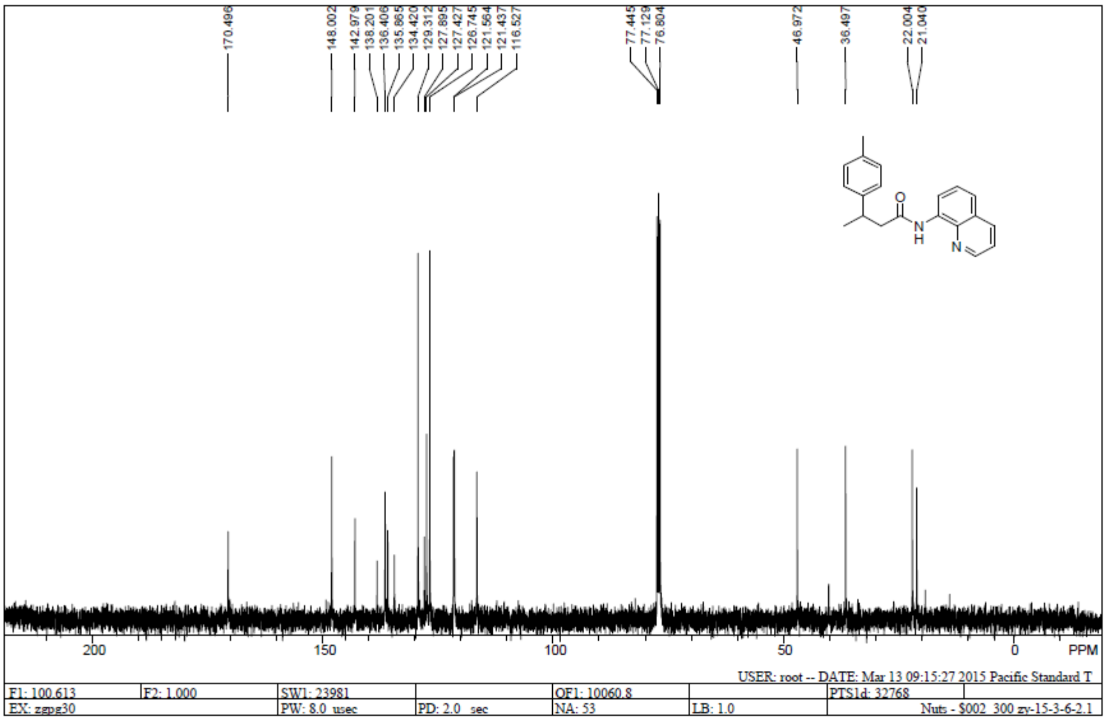

^{1}H and ^{13}C NMR spectra of 4c

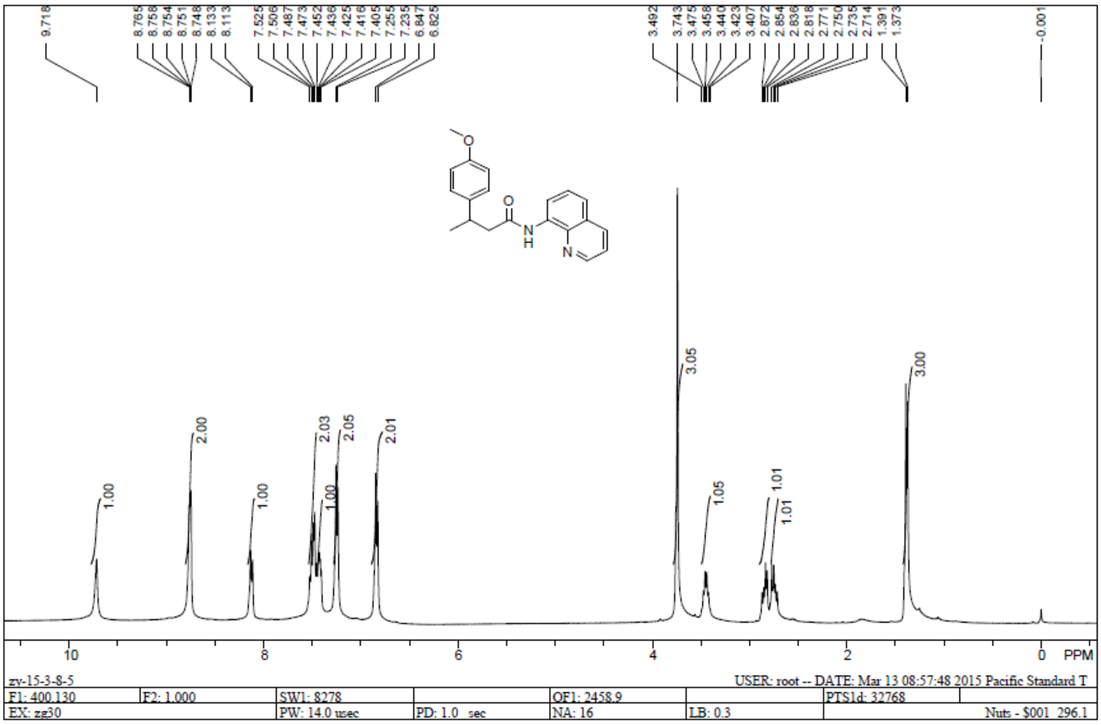

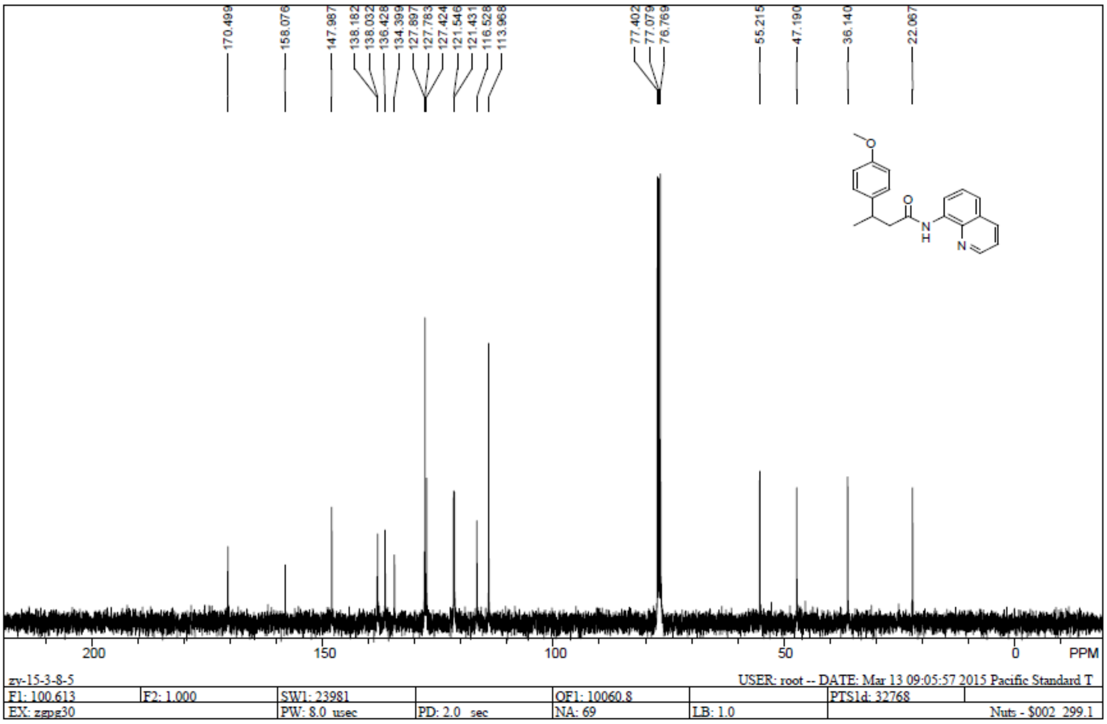

^{1}H and ^{13}C NMR spectra of 4d

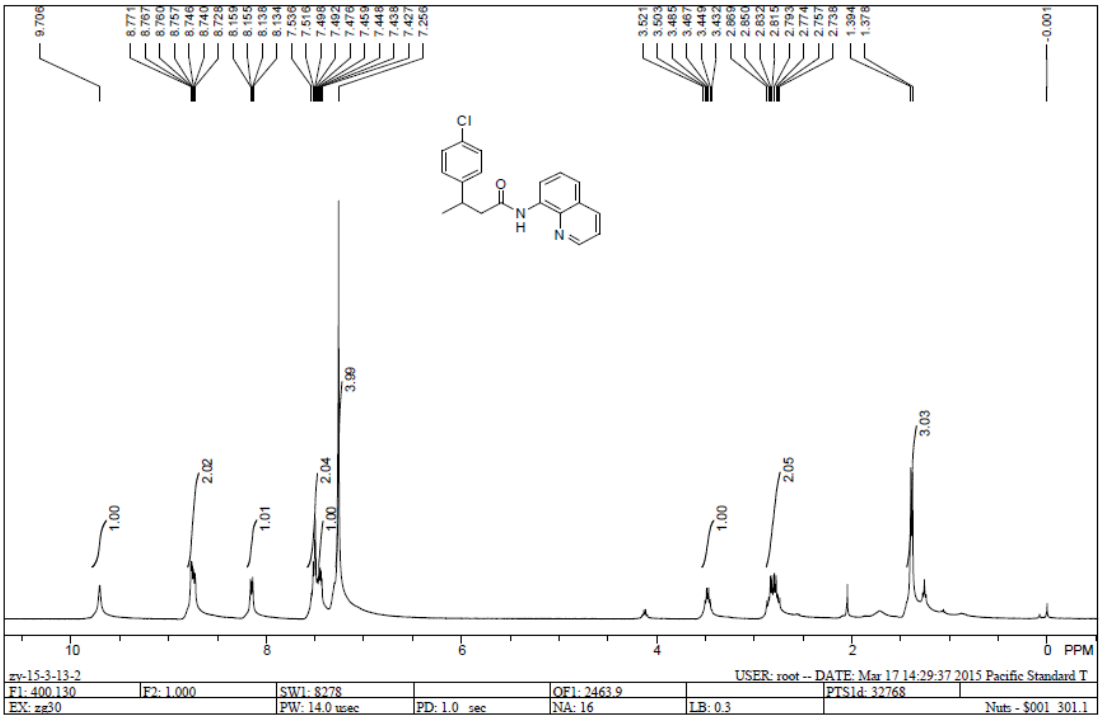

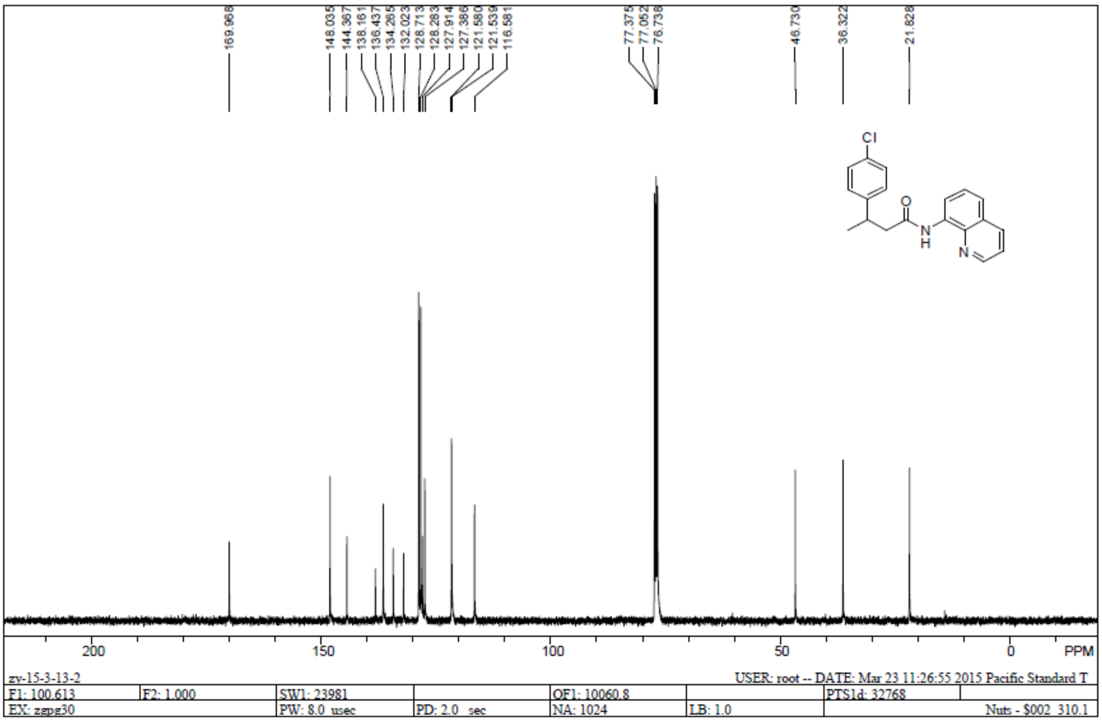

^{1}H and ^{13}C NMR spectra of 4e

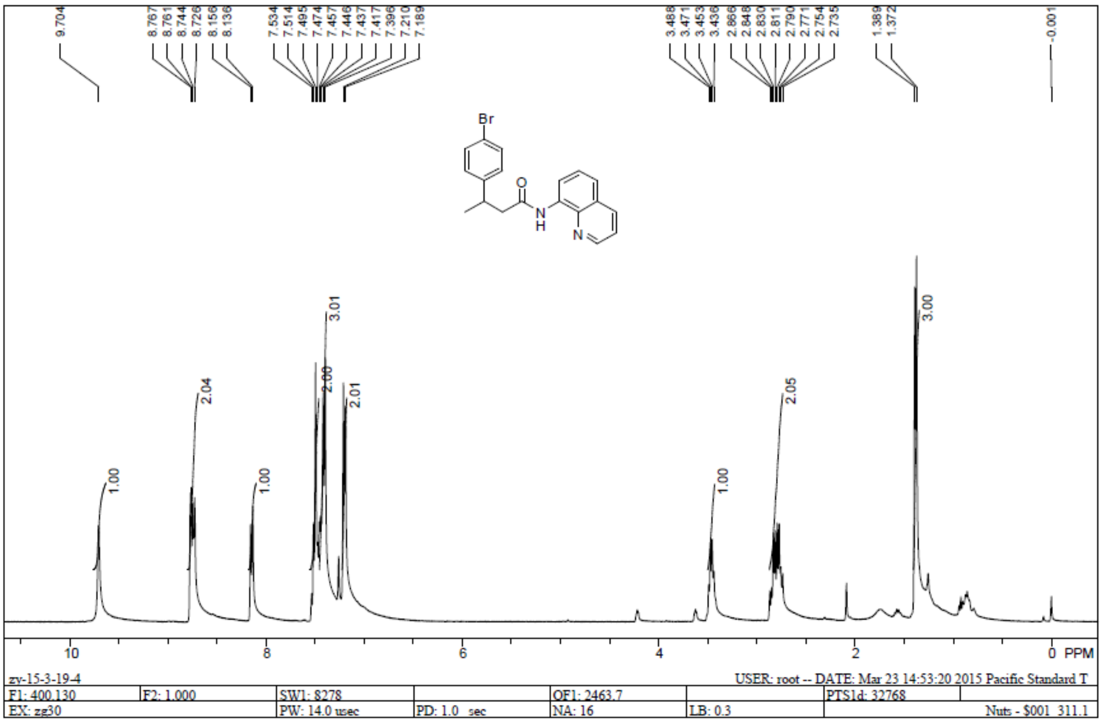

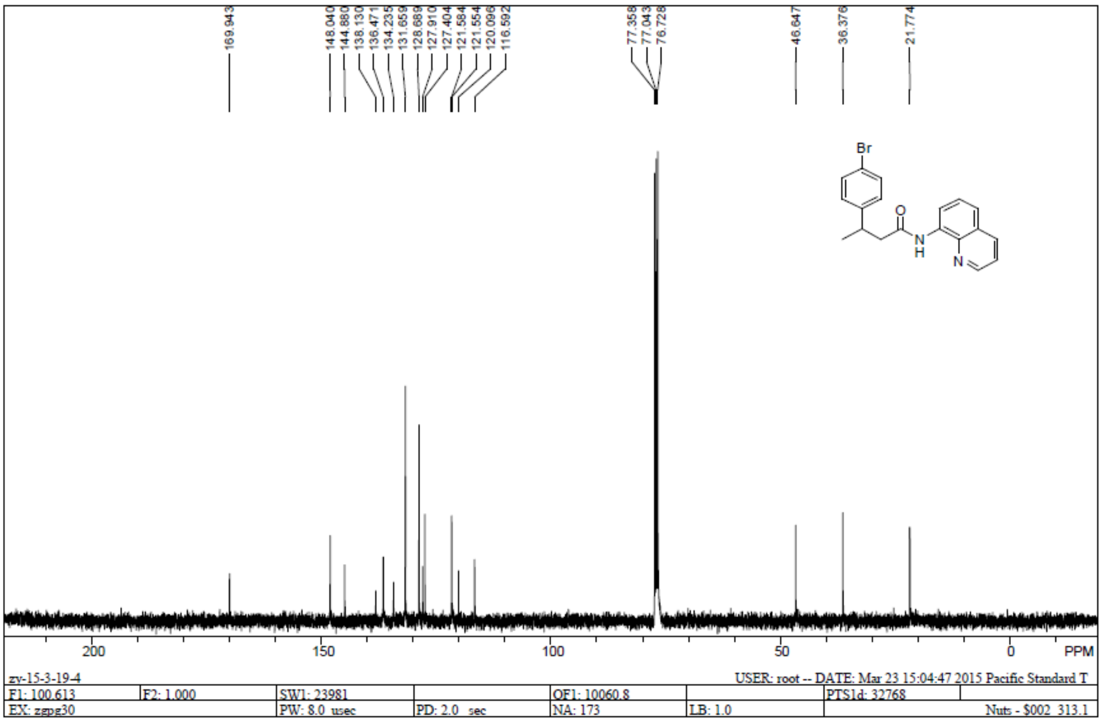

^{1}H and ^{13}C NMR spectra of 4f

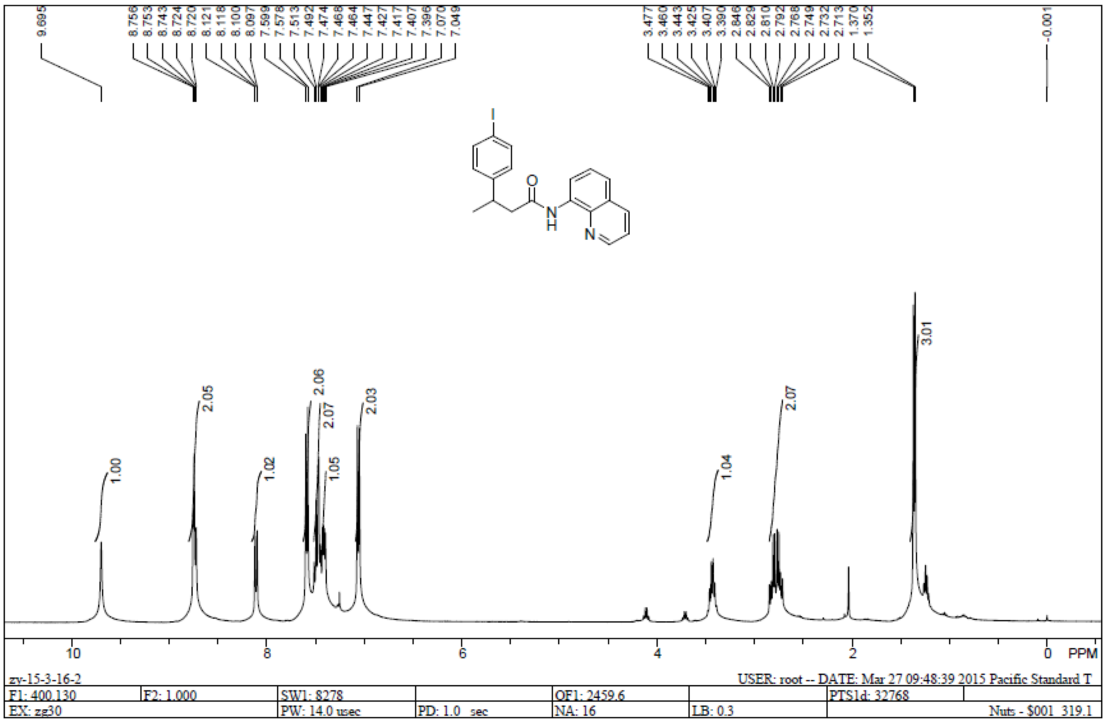

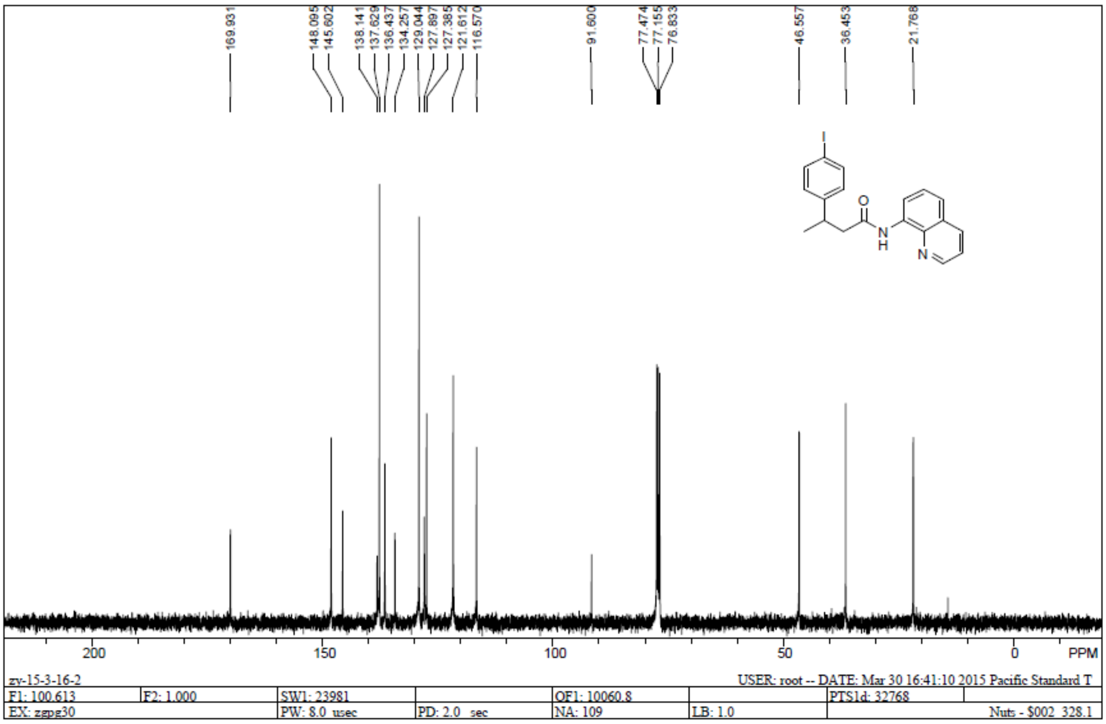

^{1}H and ^{13}C NMR spectra of 4g

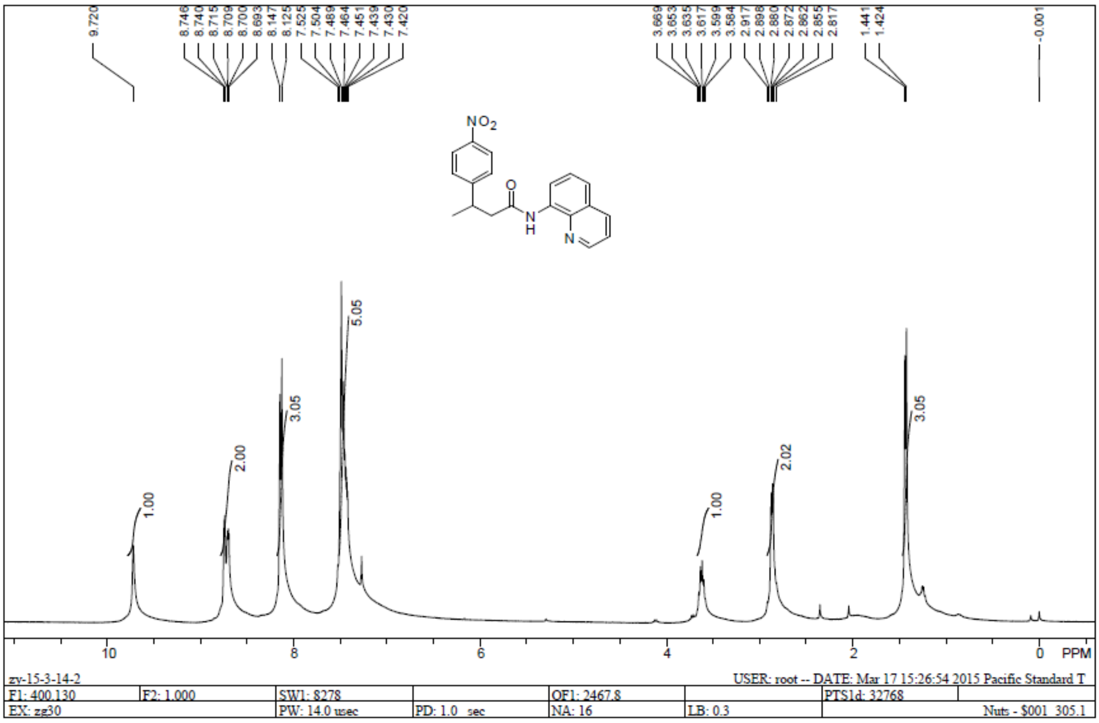

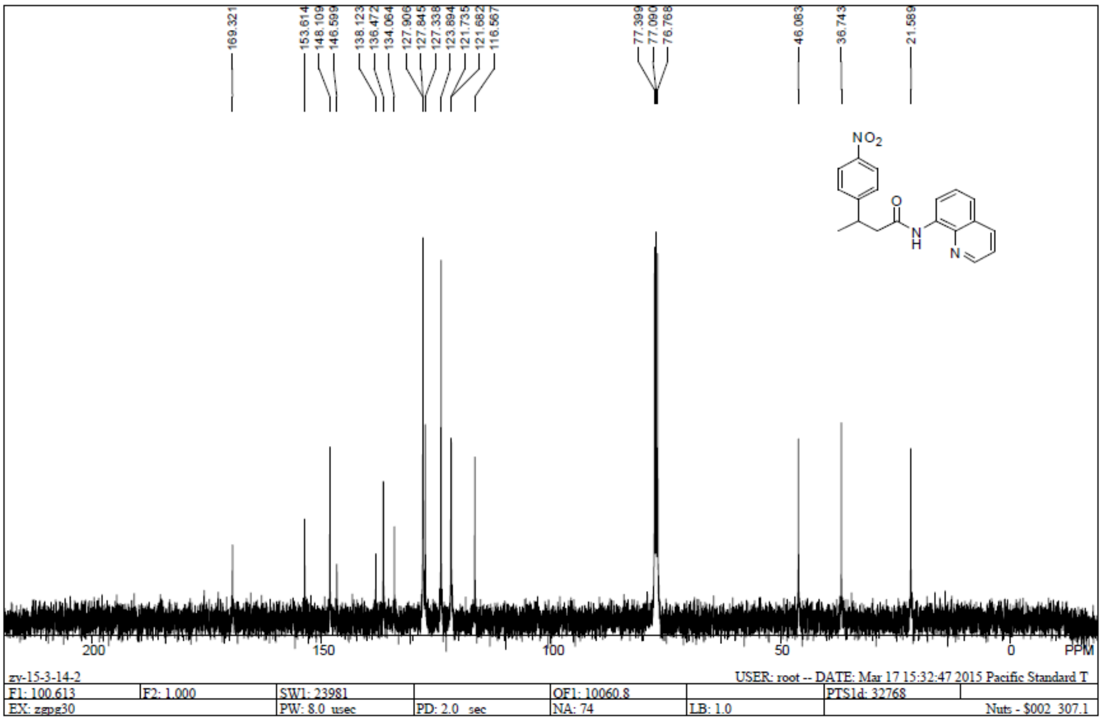

^{1}H and ^{13}C NMR spectra of 4h

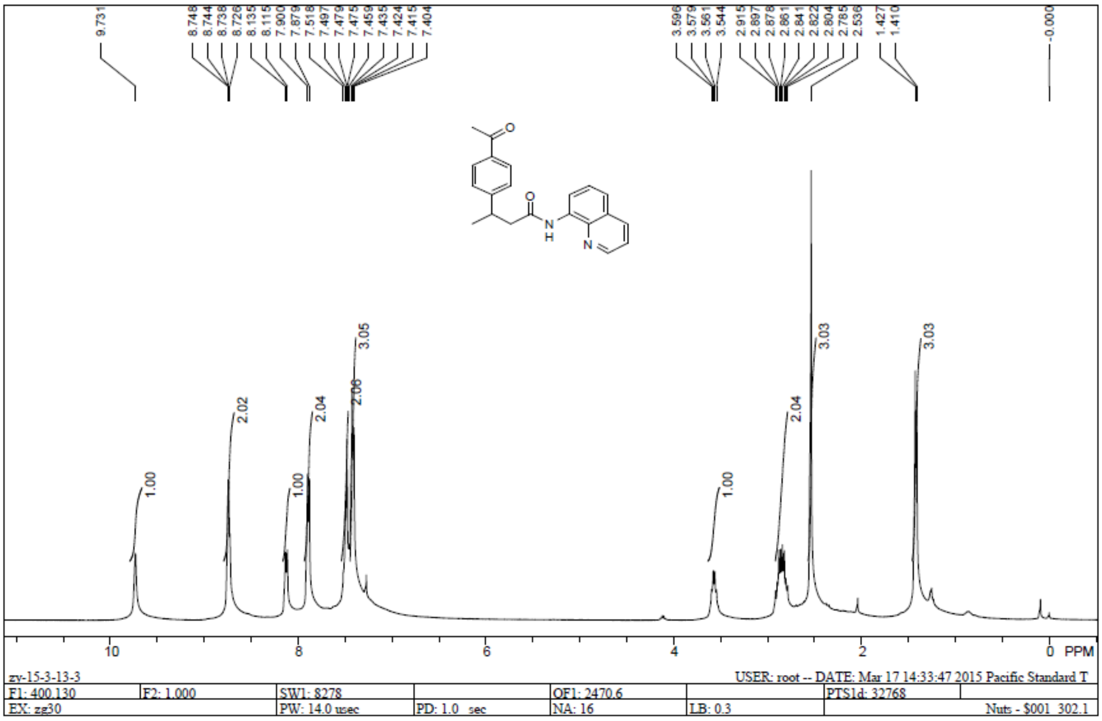

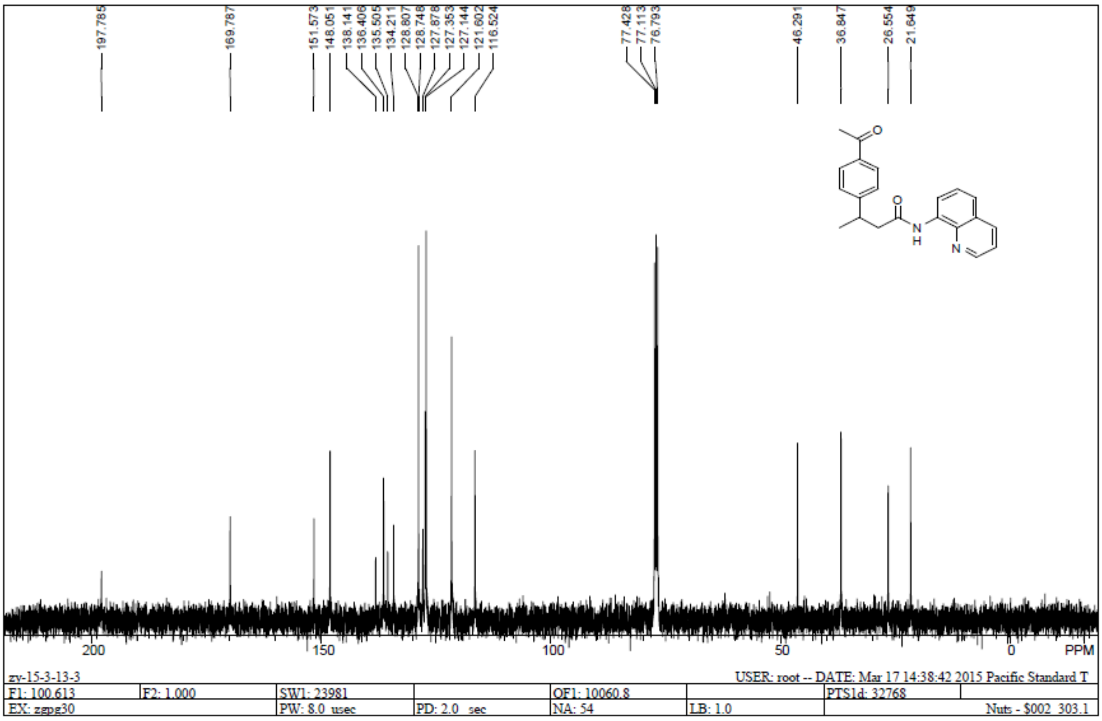

^{1}H and ^{13}C NMR spectra of 4i

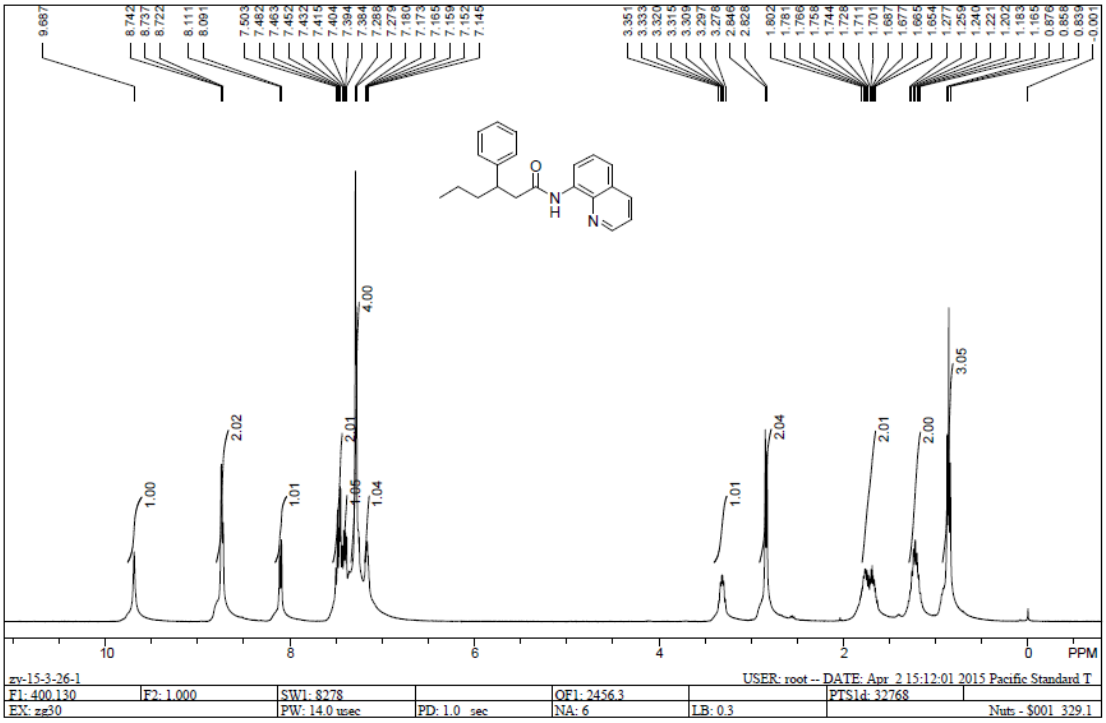

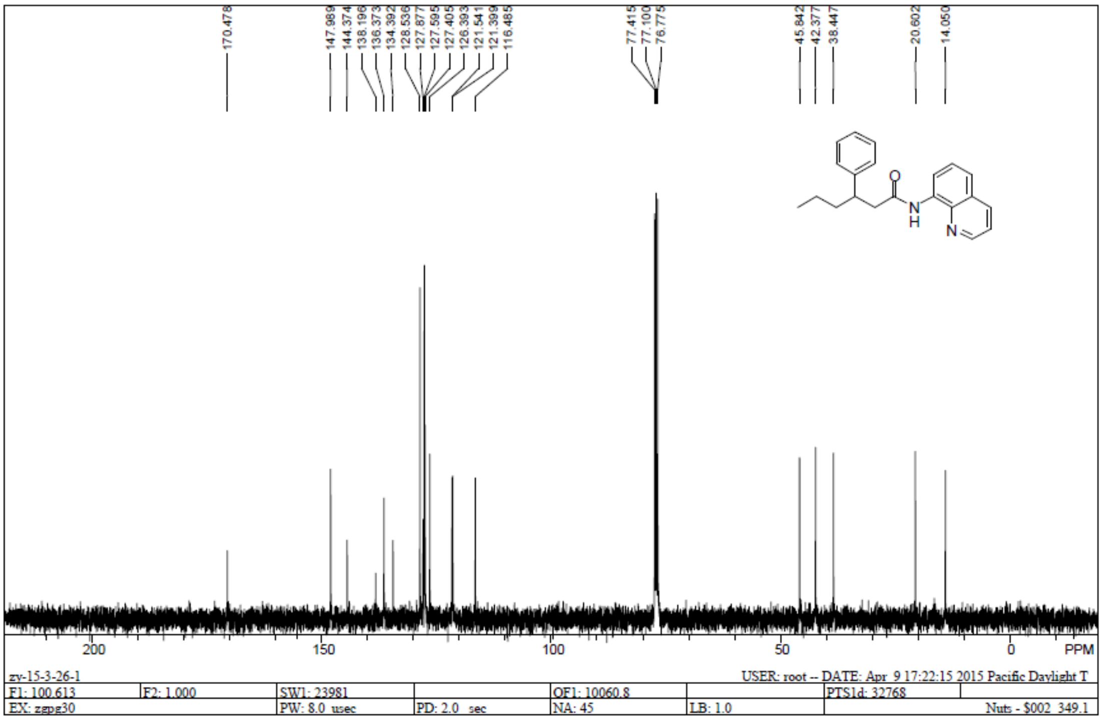

^{1}H and ^{13}C NMR spectra of 4j

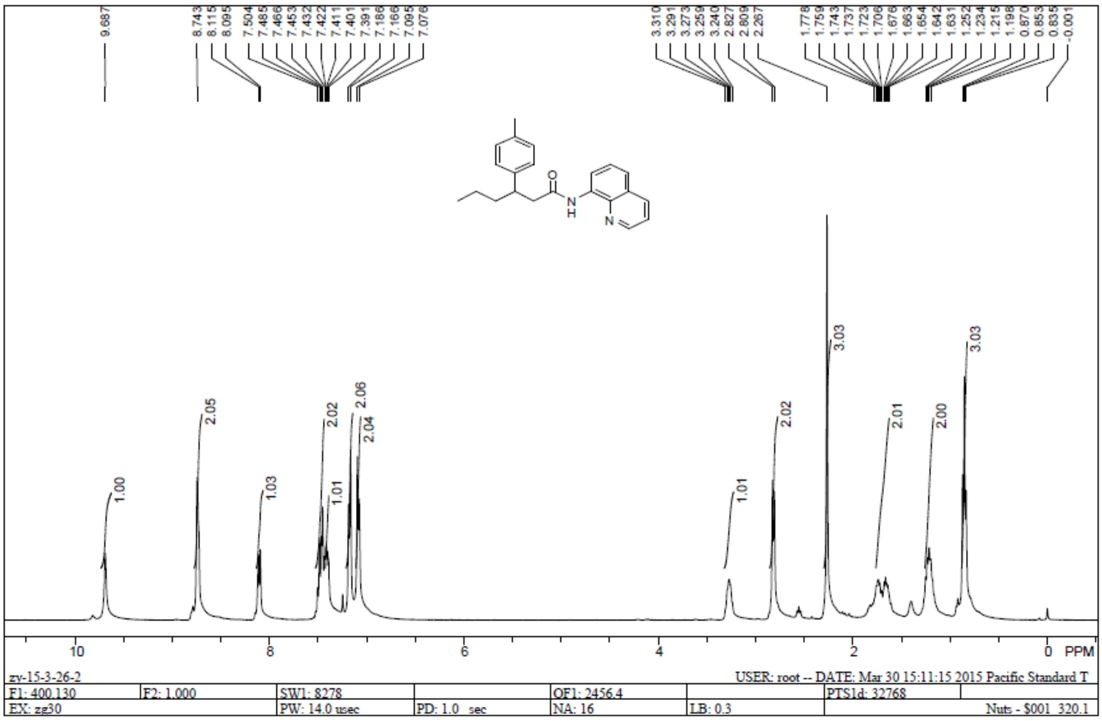

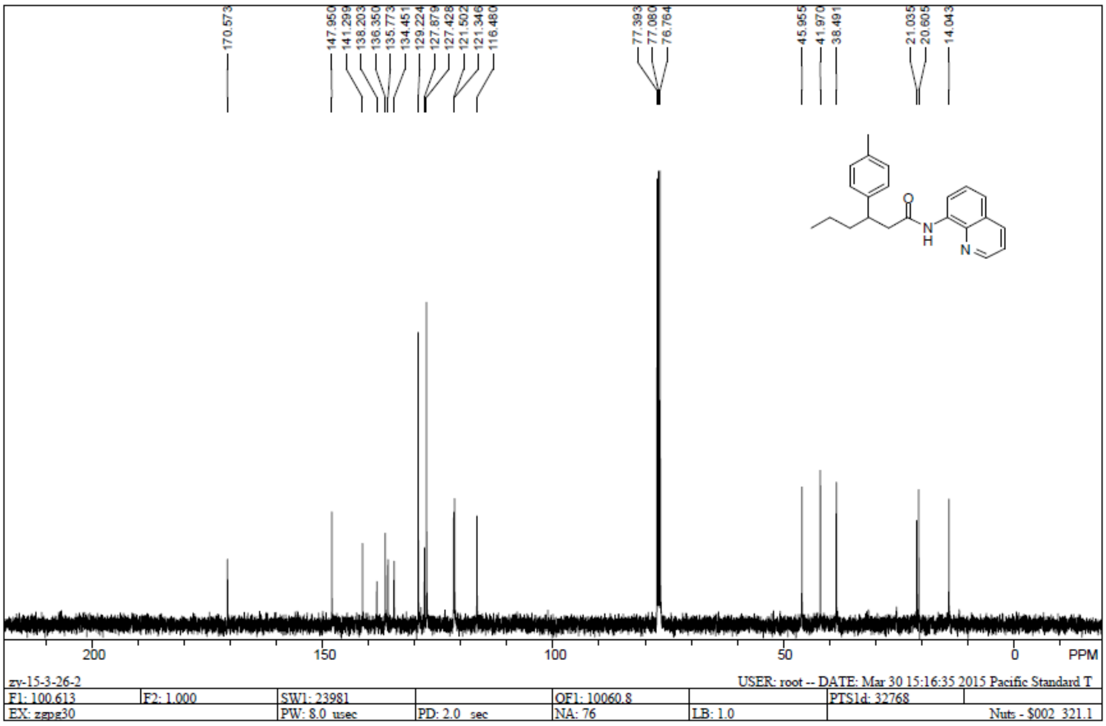

^{1}H and ^{13}C NMR spectra of 4k

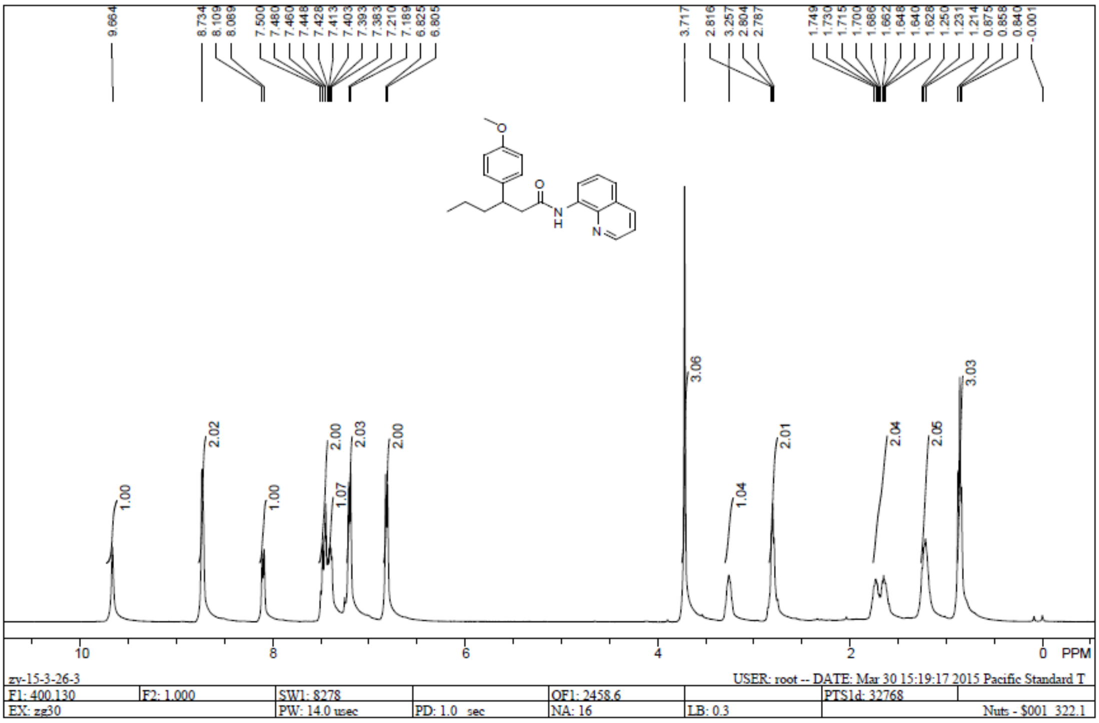

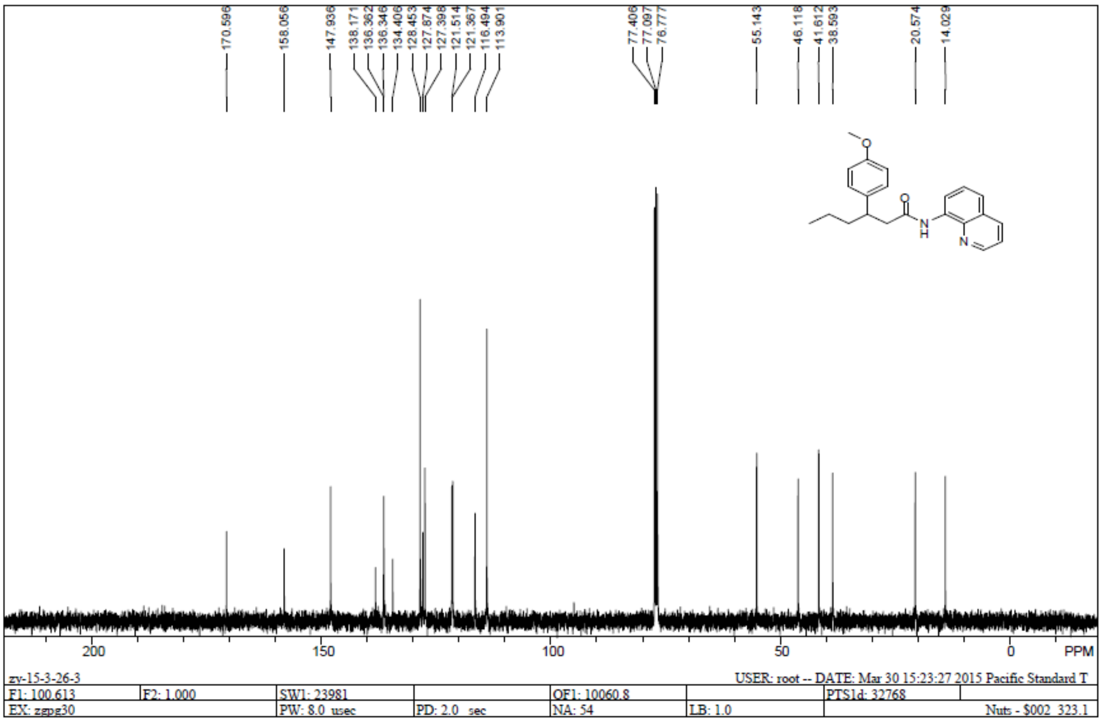

^{1}H and ^{13}C NMR spectra of 4l

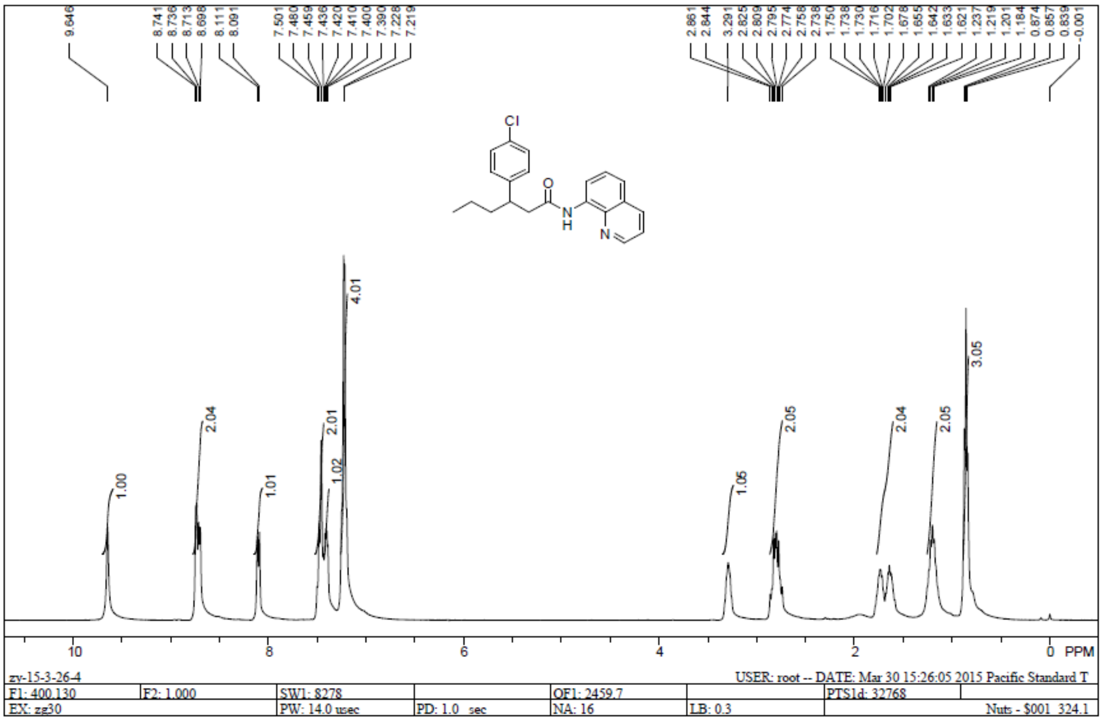

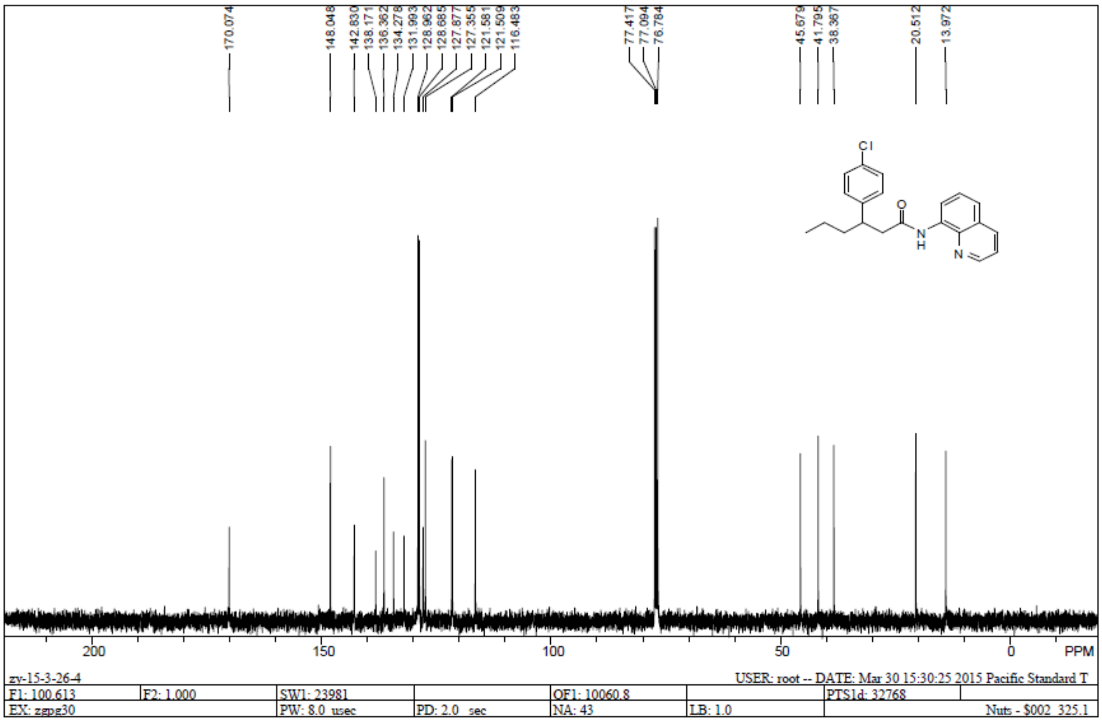

^{1}H and ^{13}C NMR of 4m

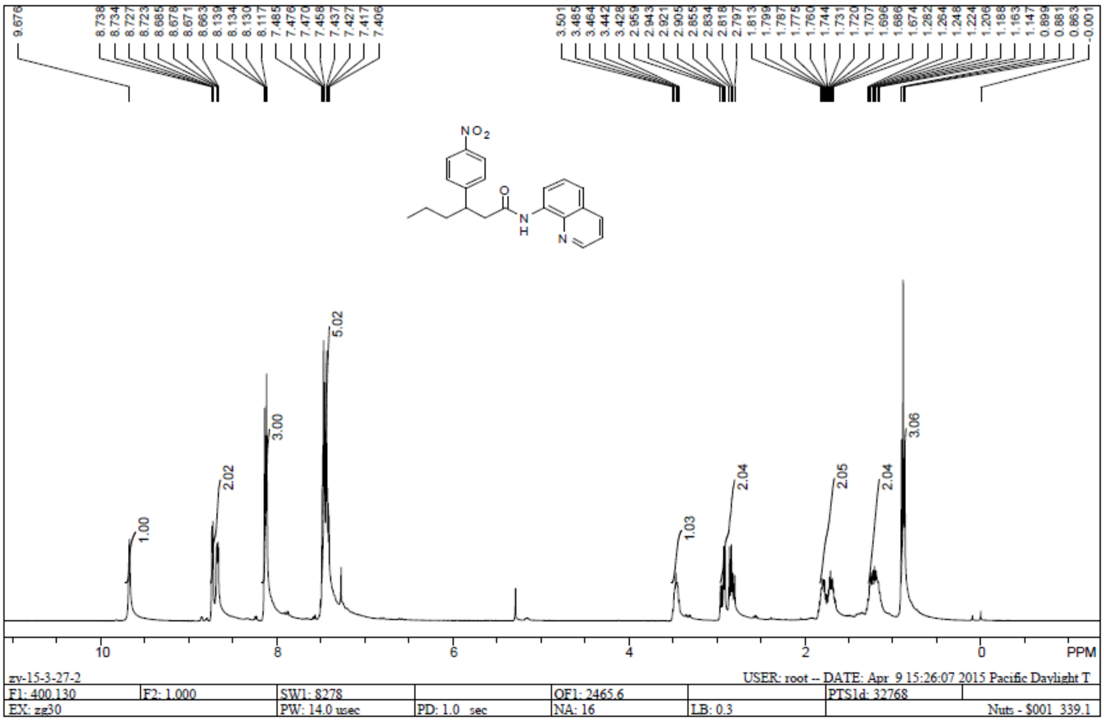

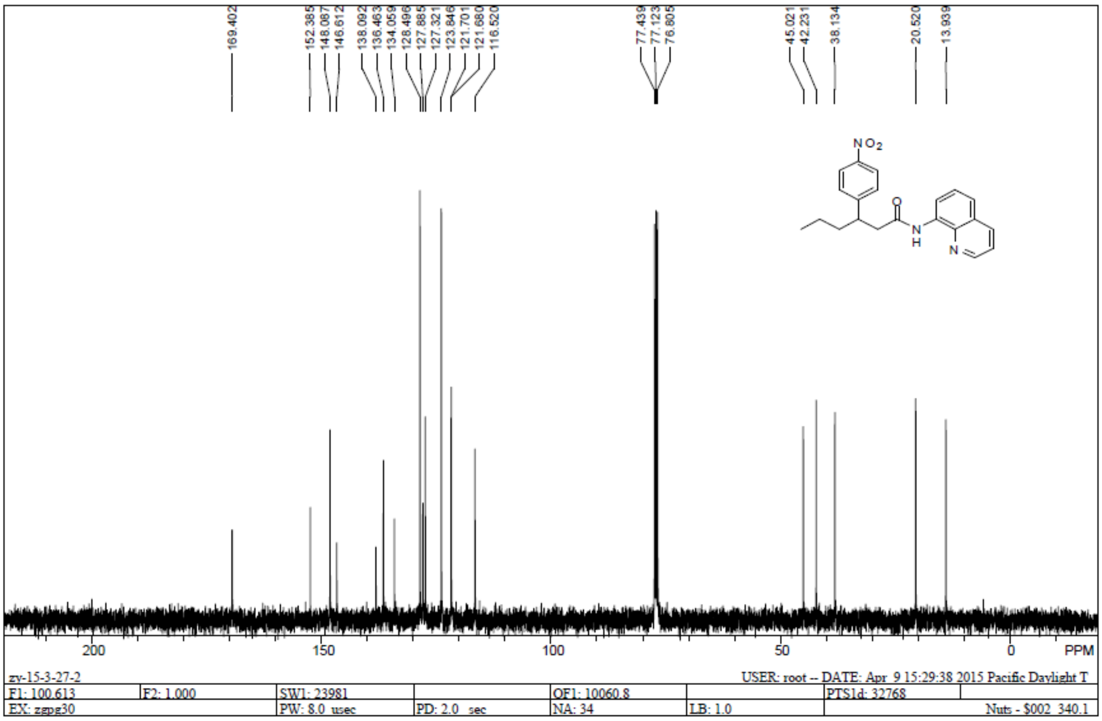

^{1}H and ^{13}C NMR spectra of 4n

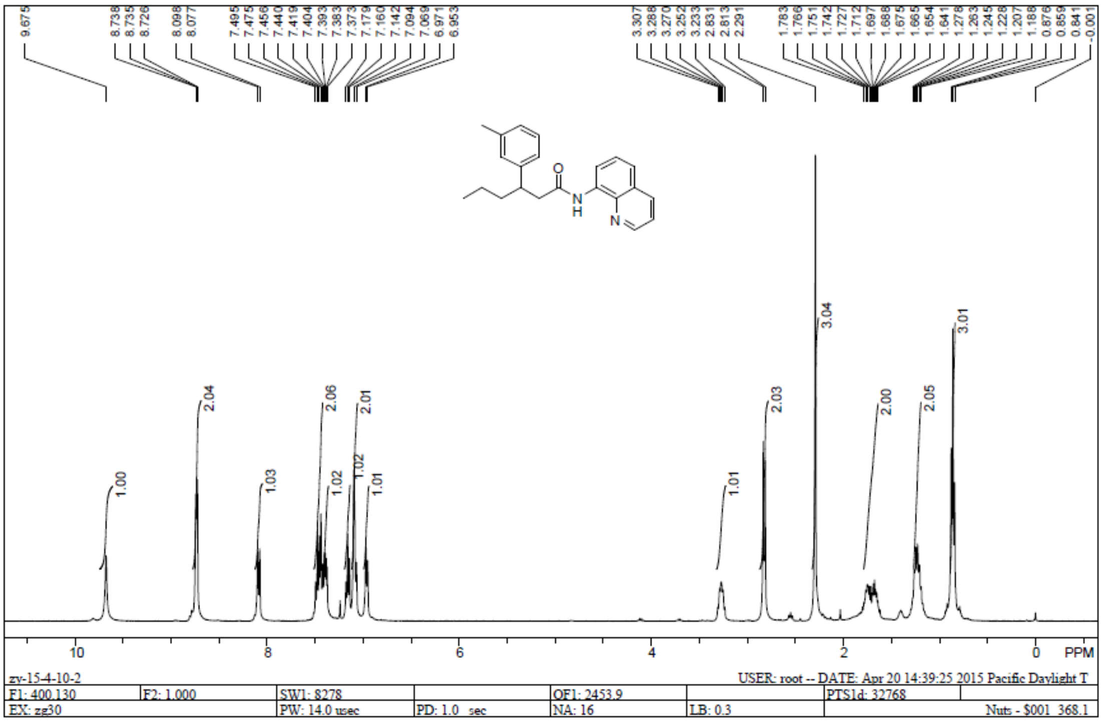

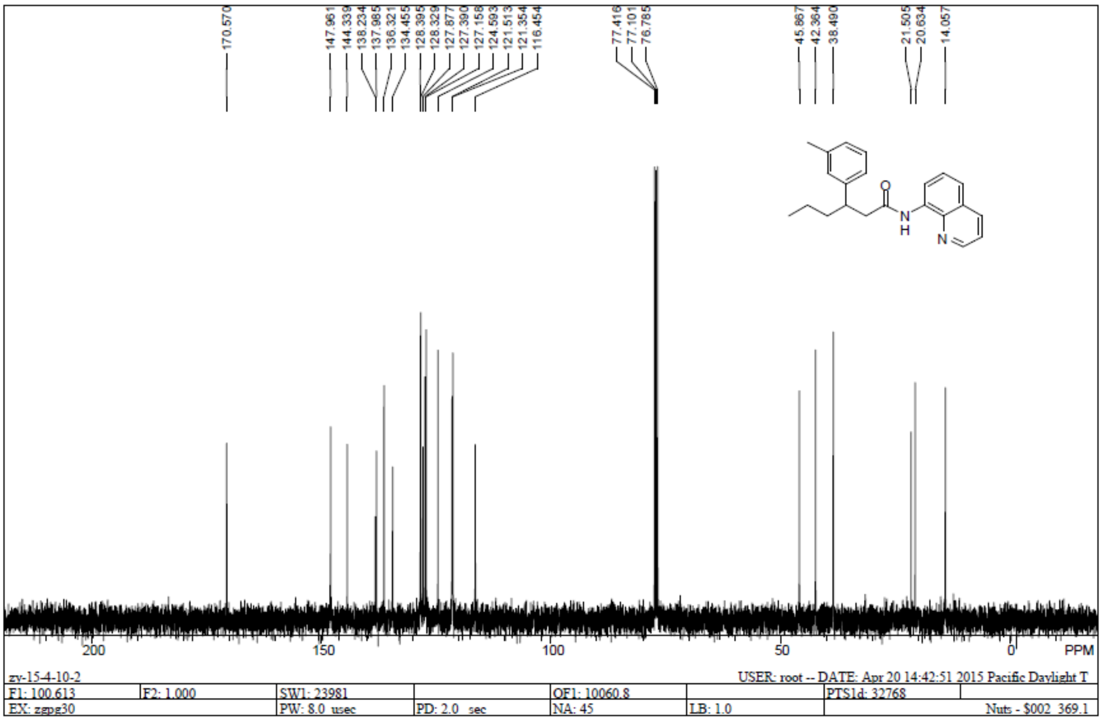

^{1}H and ^{13}C NMR spectra of 4o

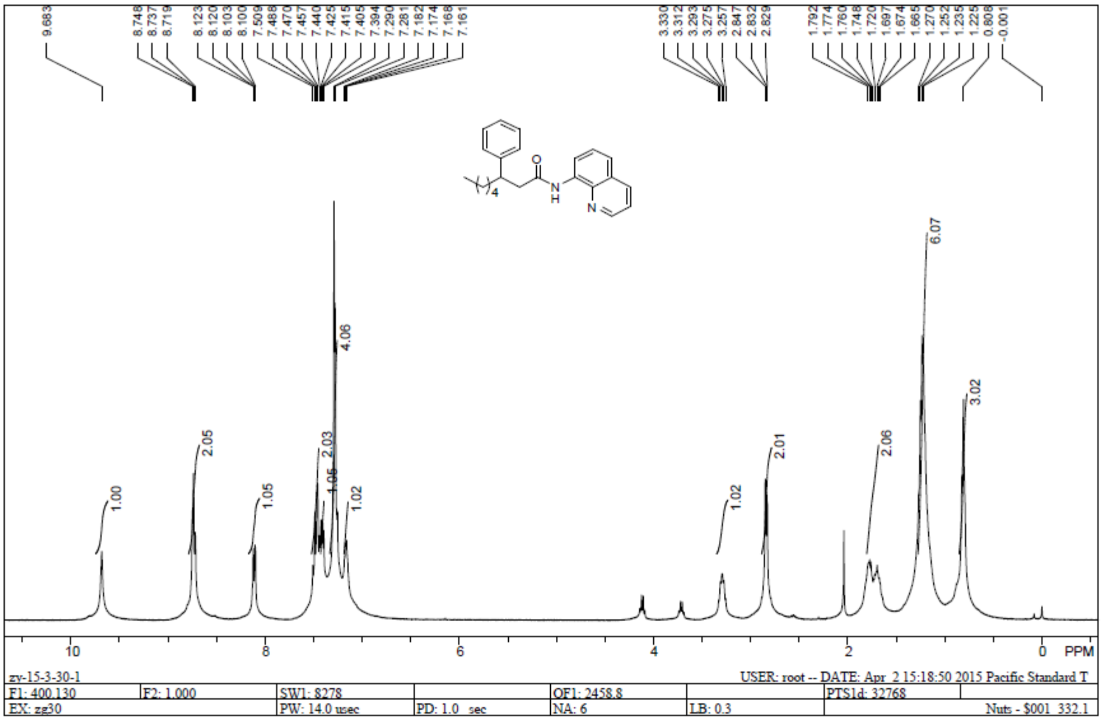

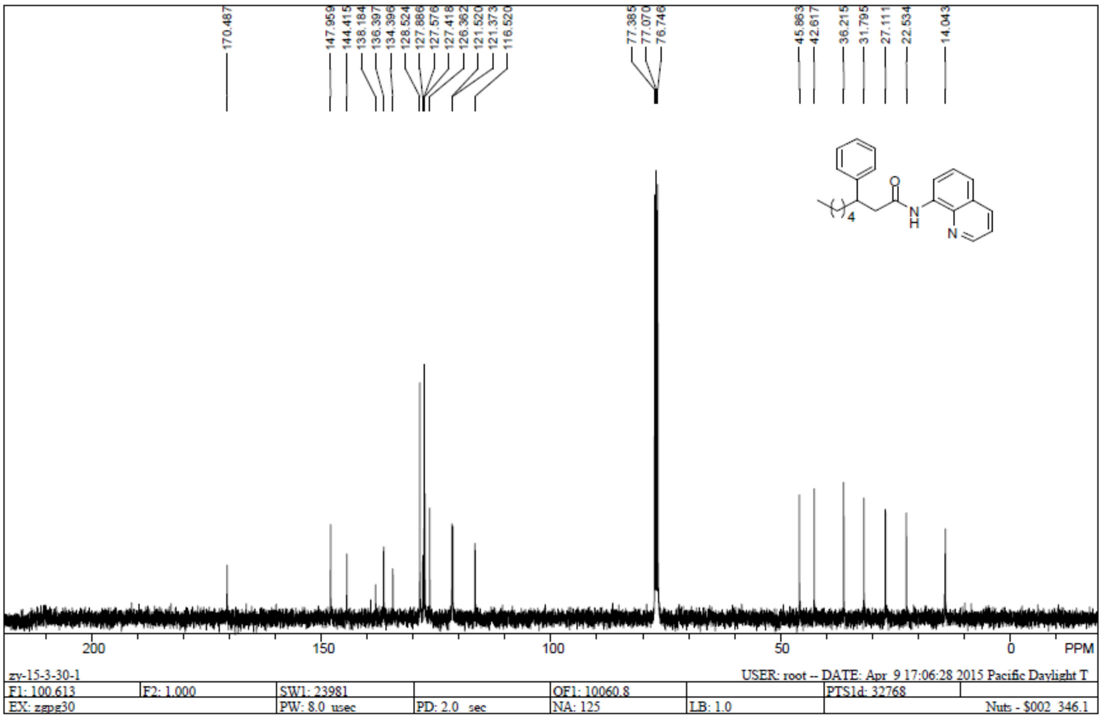

^{1}H and ^{13}C NMR spectra of 4p

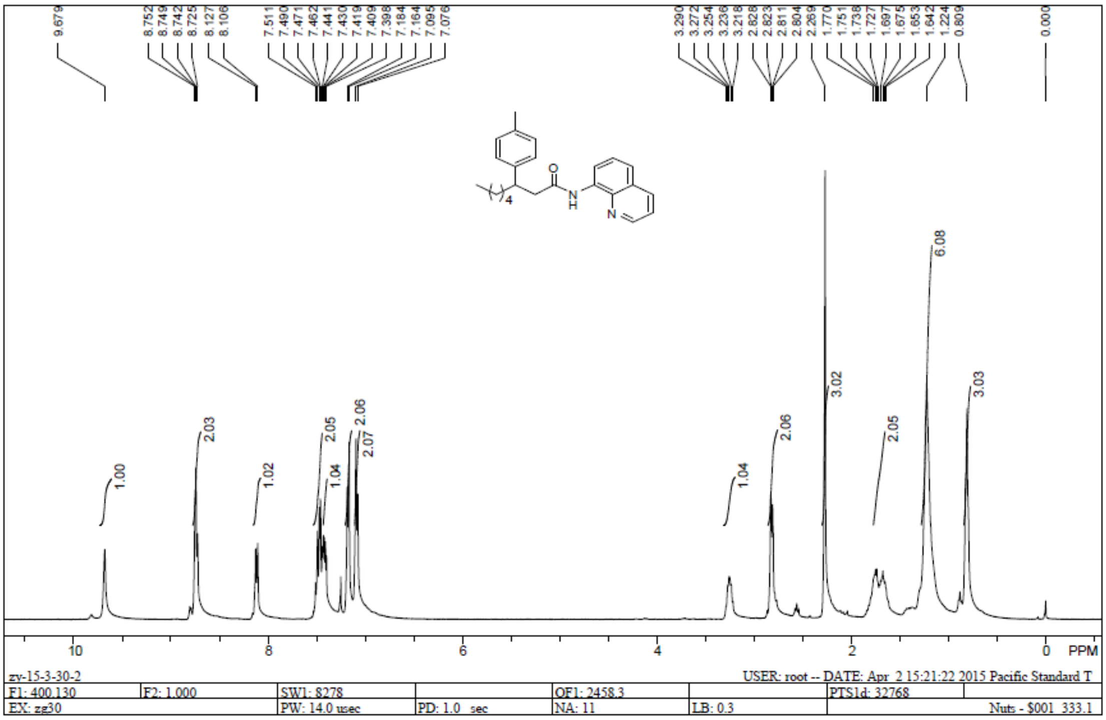

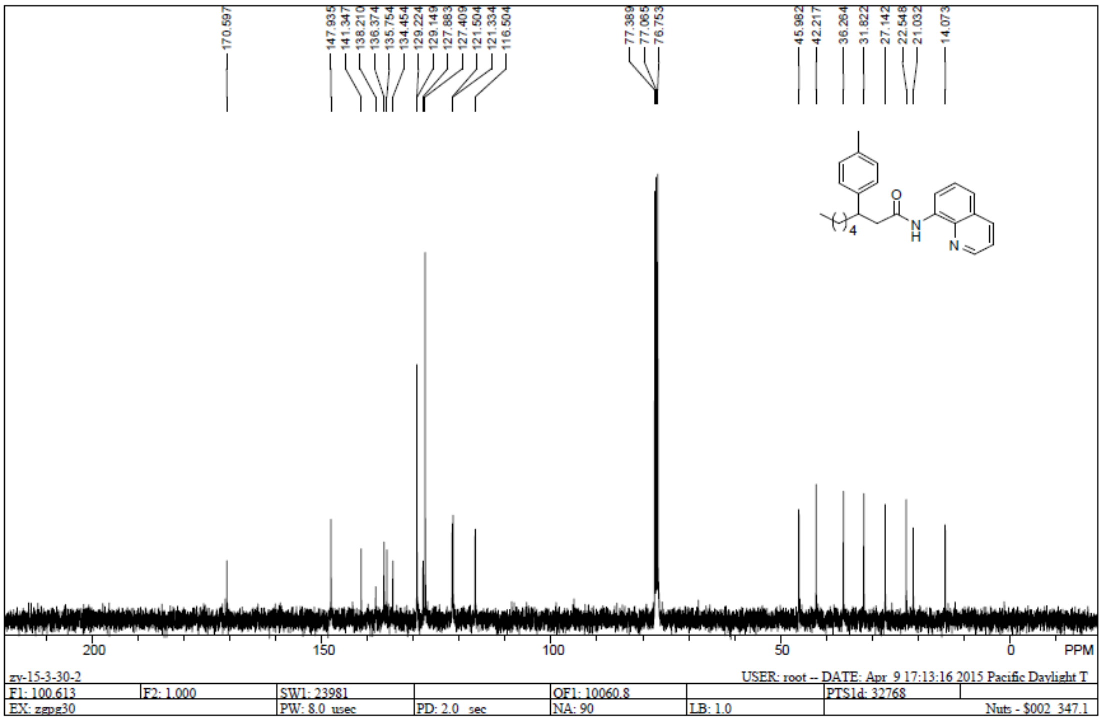

^{1}H and ^{13}C NMR spectra of 4q

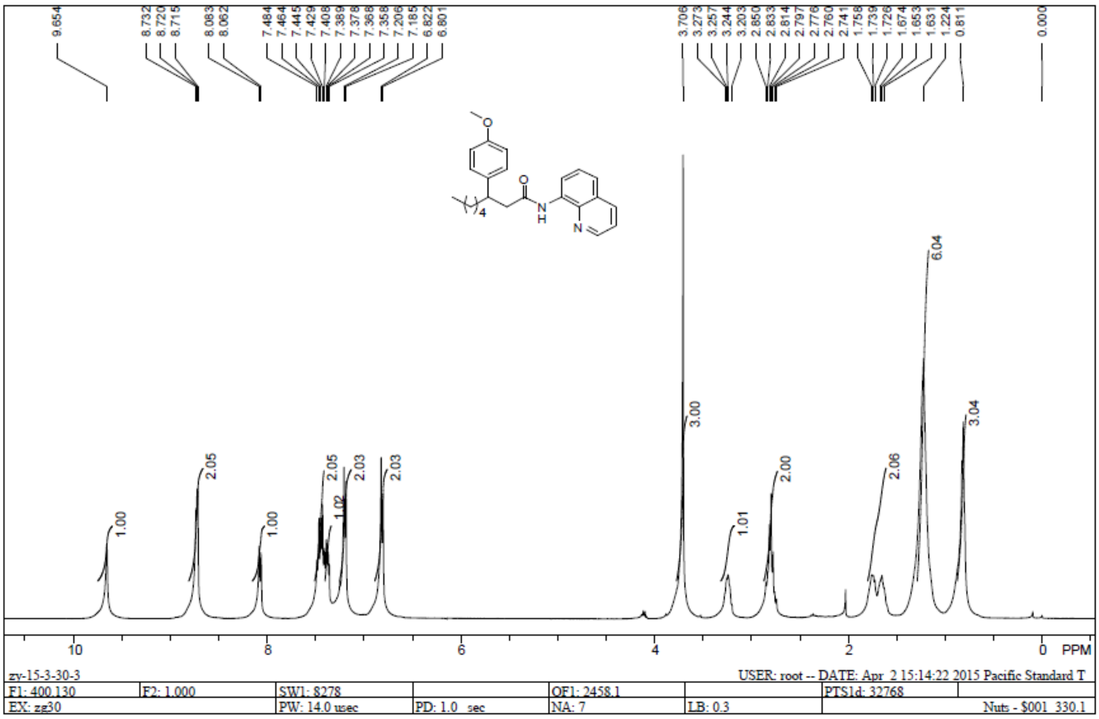

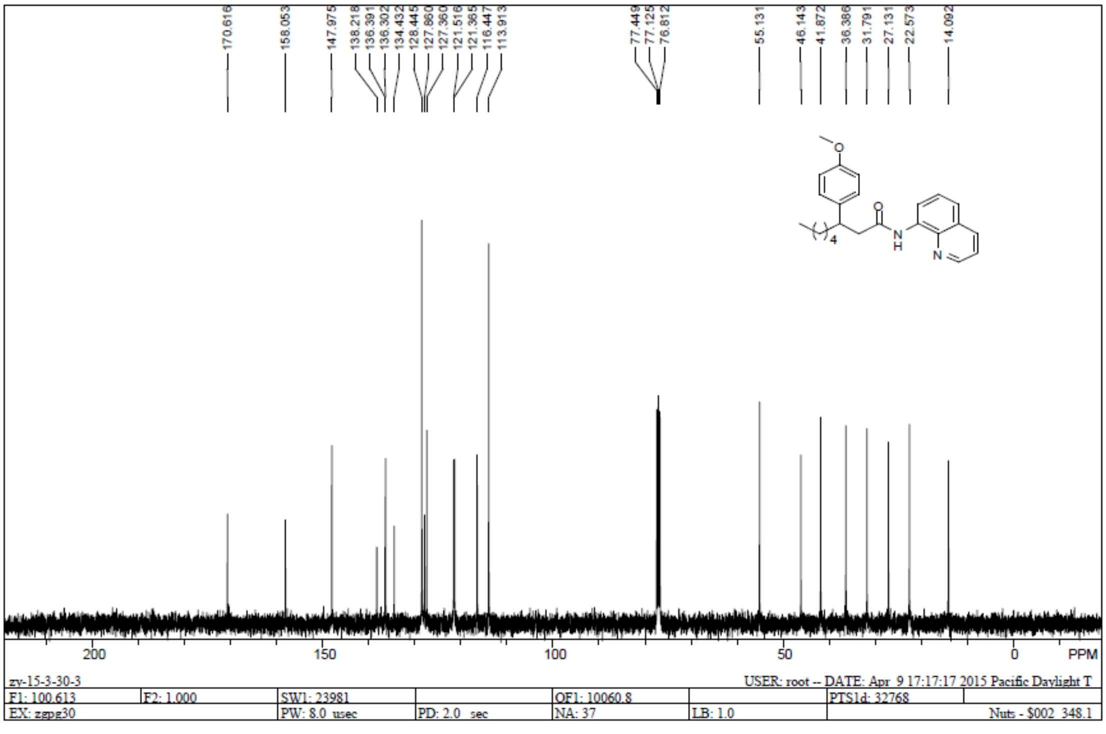

^{1}H and ^{13}C NMR spectra of 4r

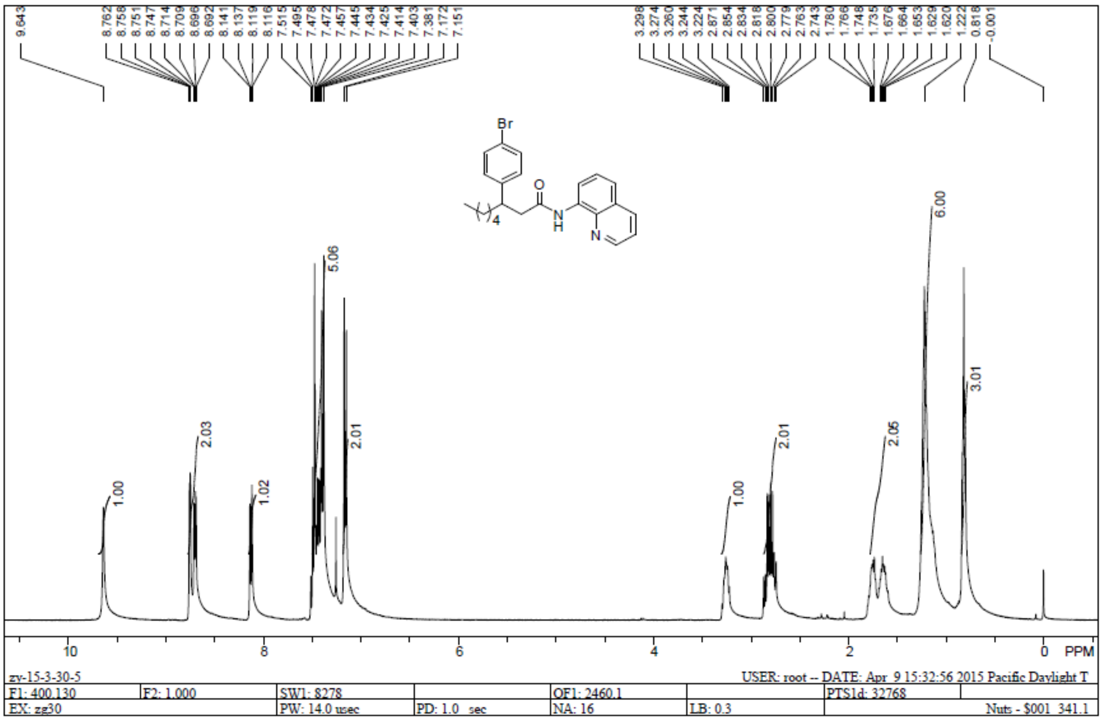

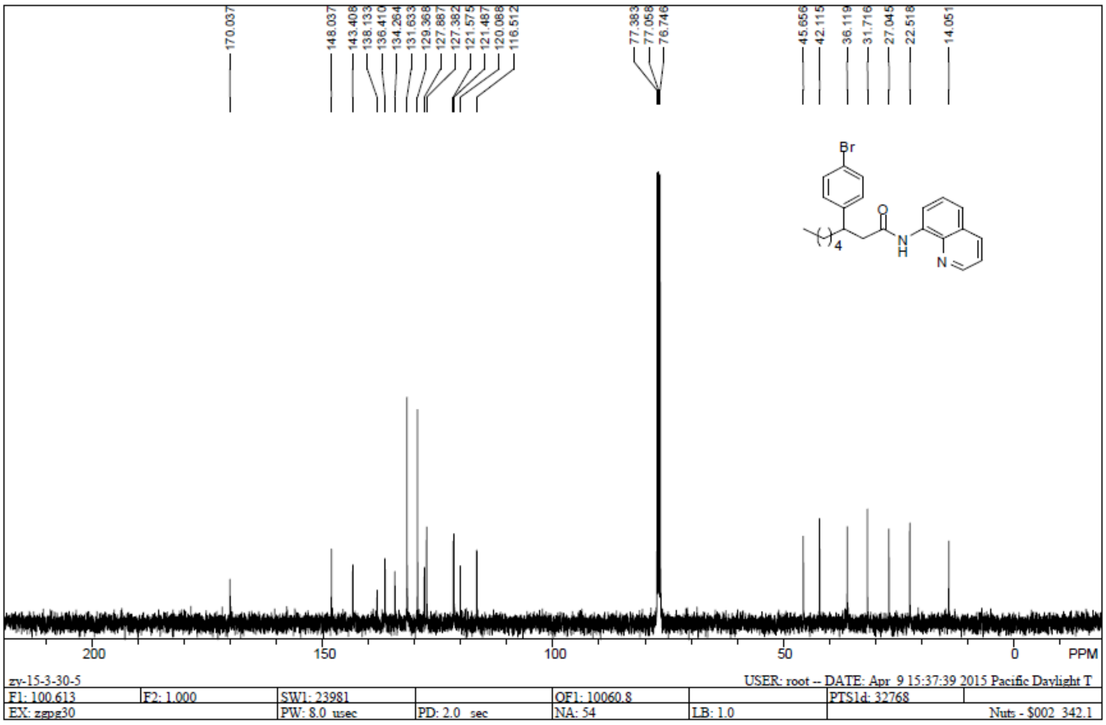

^{1}H and ^{13}C NMR spectra of 4s

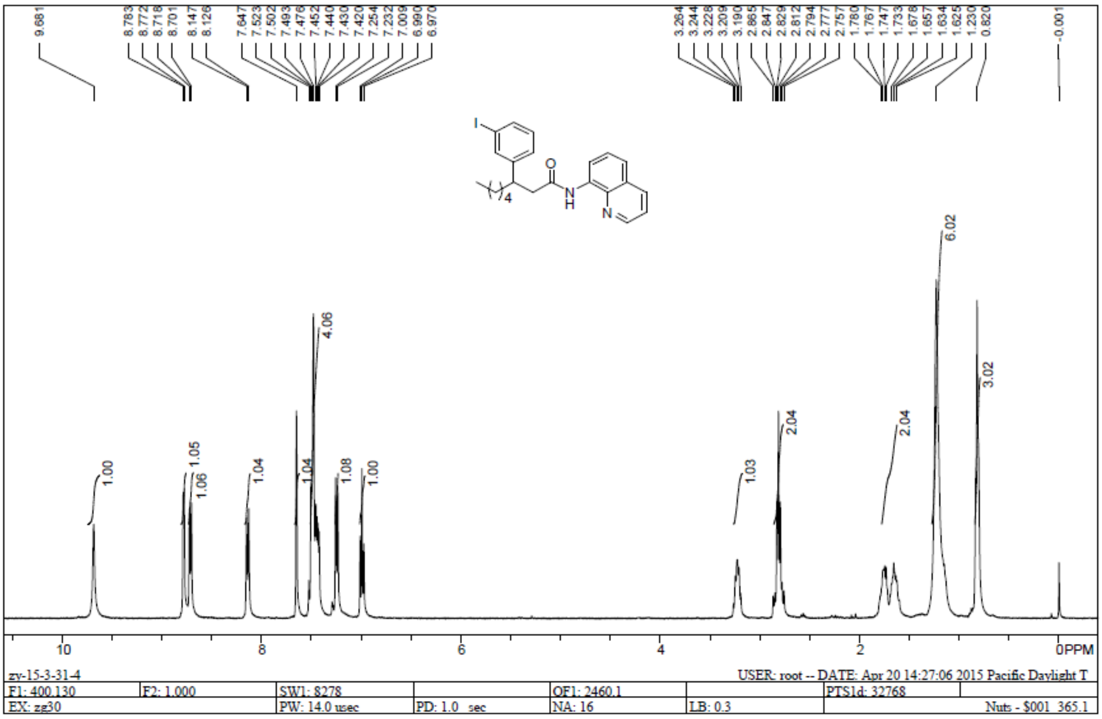

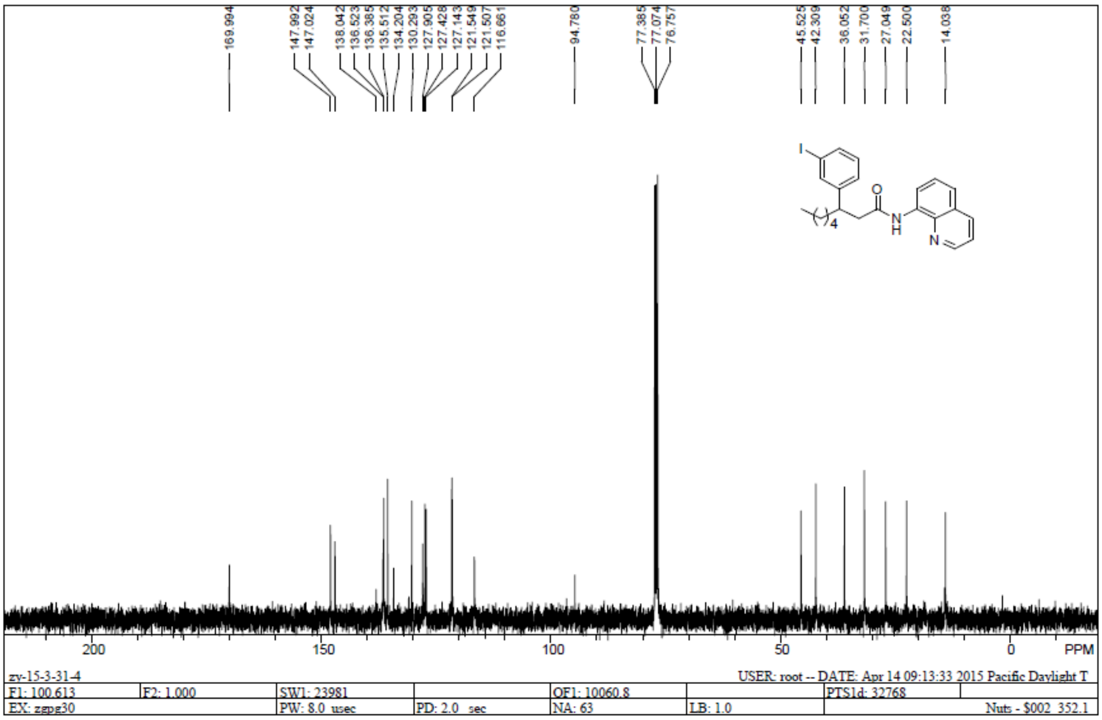

^{1}H and ^{13}C NMR spectra of 4t

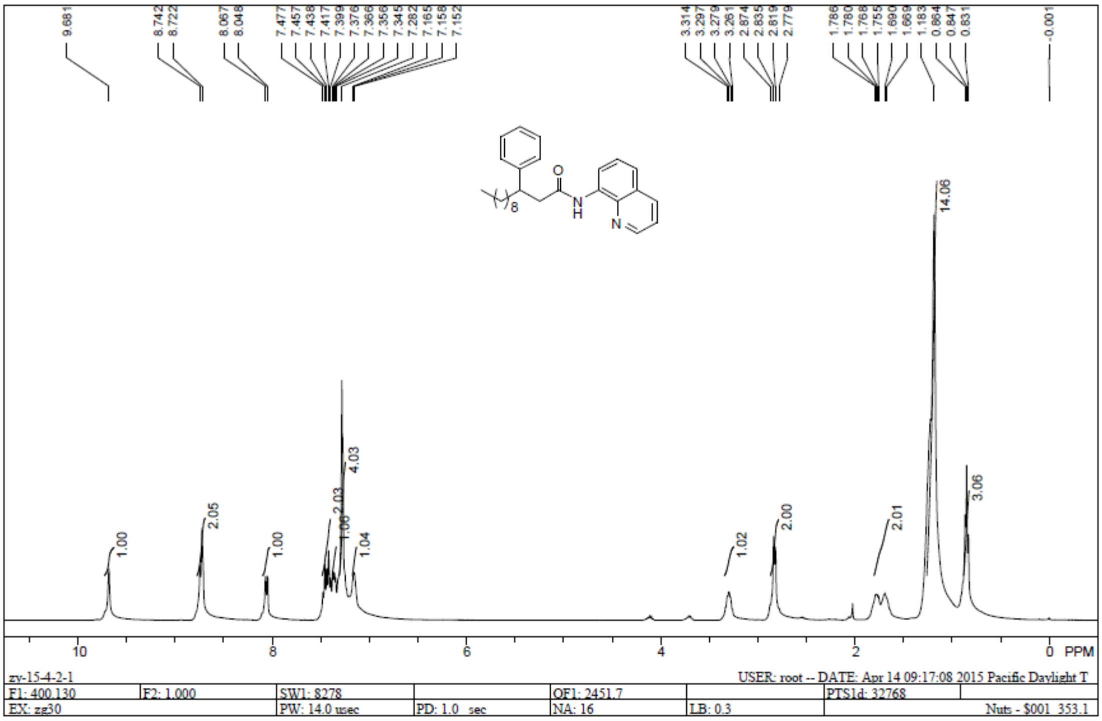

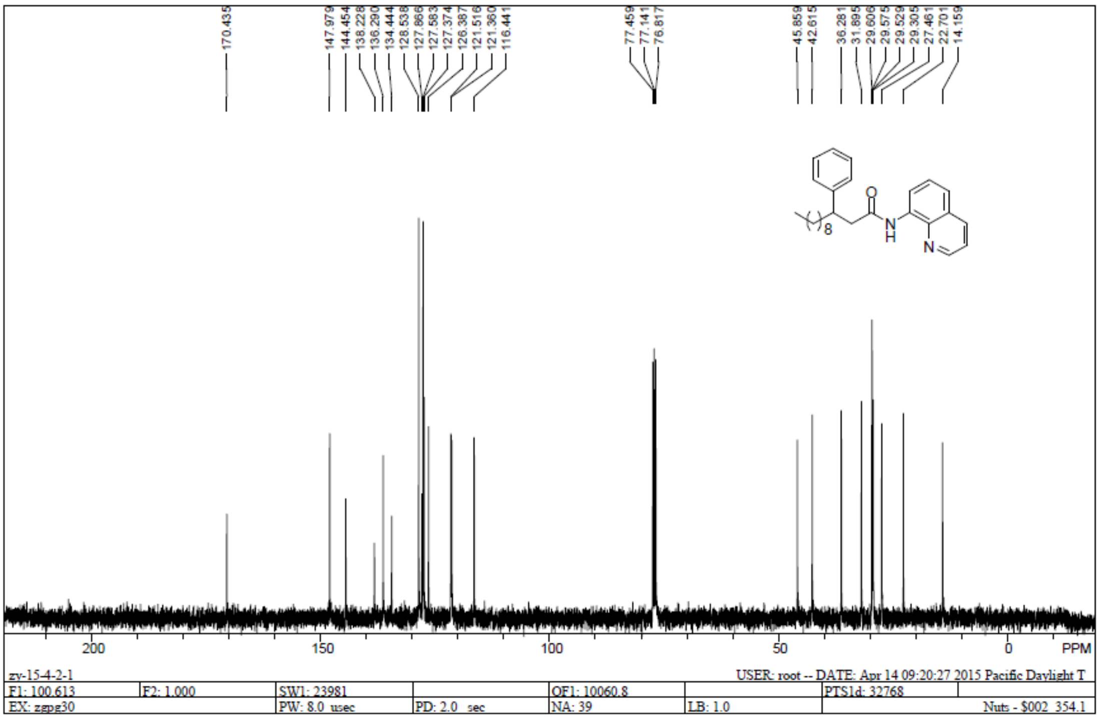

^{1}H and ^{13}C NMR spectra of 4u

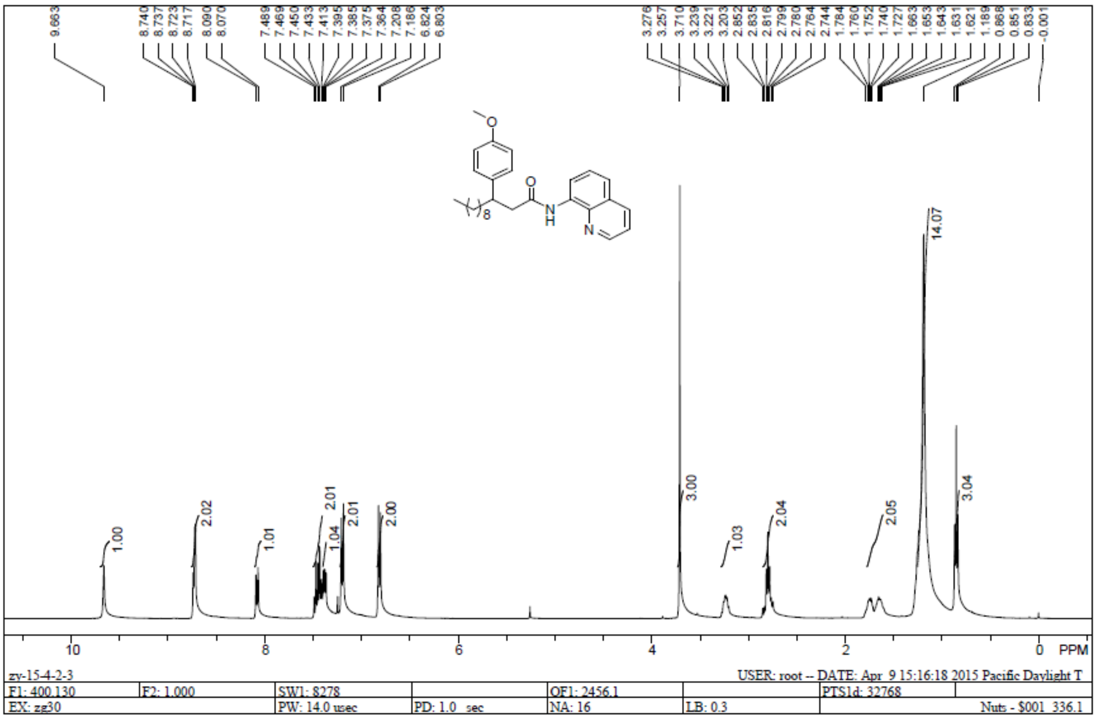

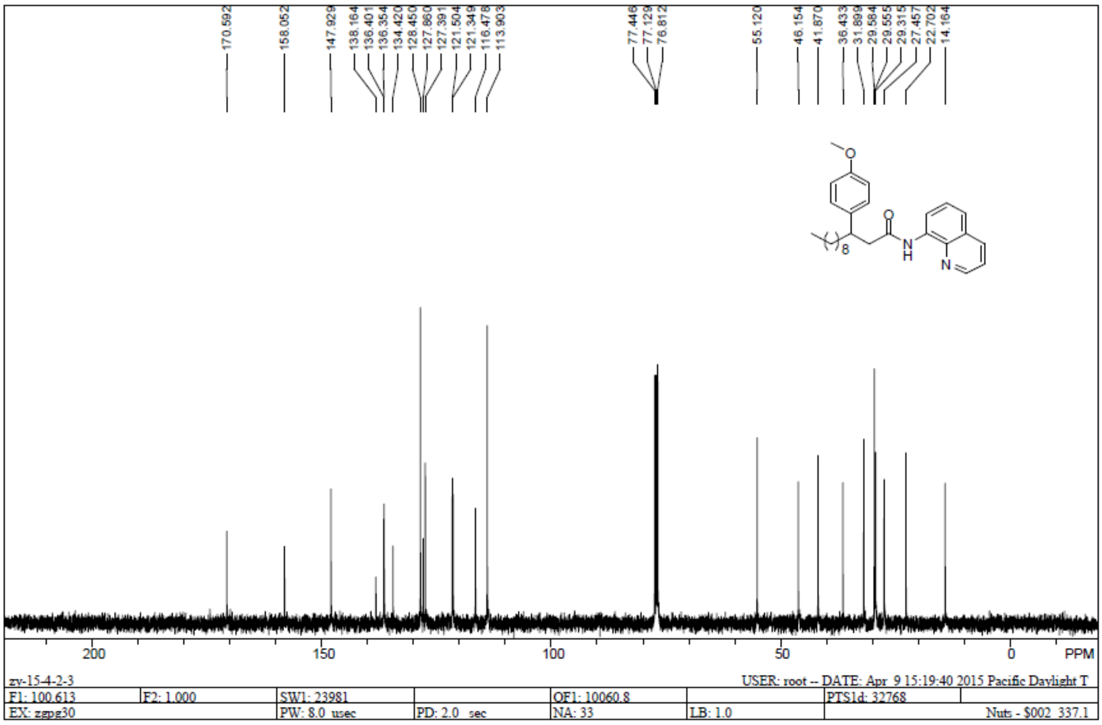

^{1}H and ^{13}C NMR spectra of 4v

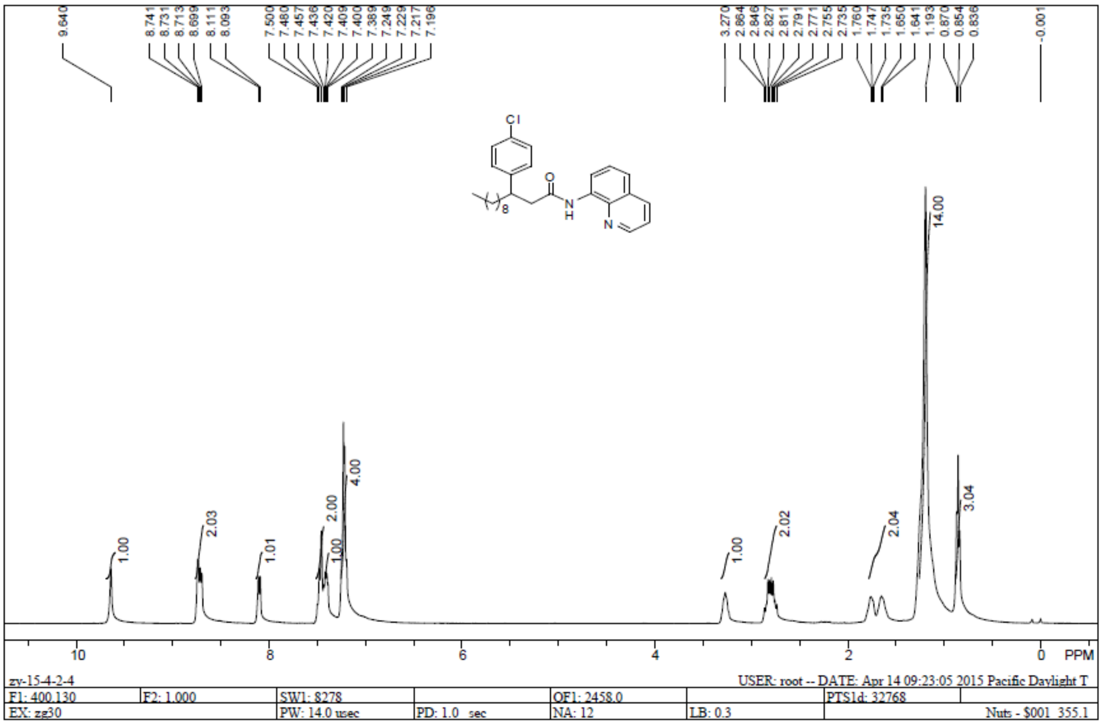

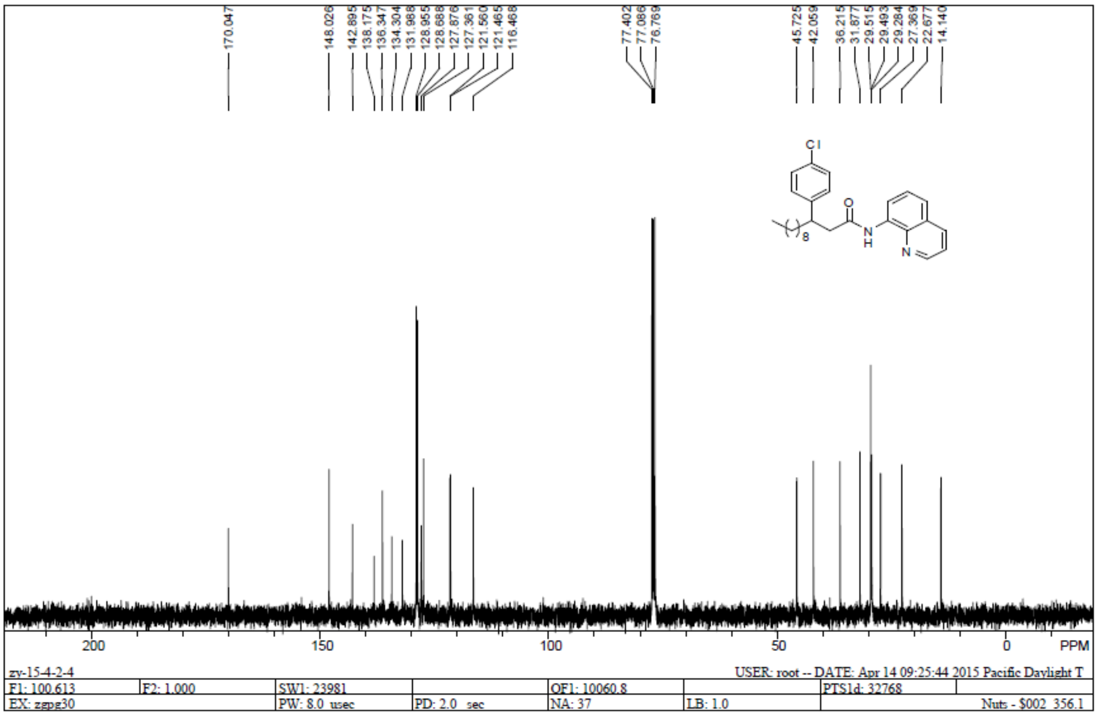

^{1}H and ^{13}C NMR spectra of 4w

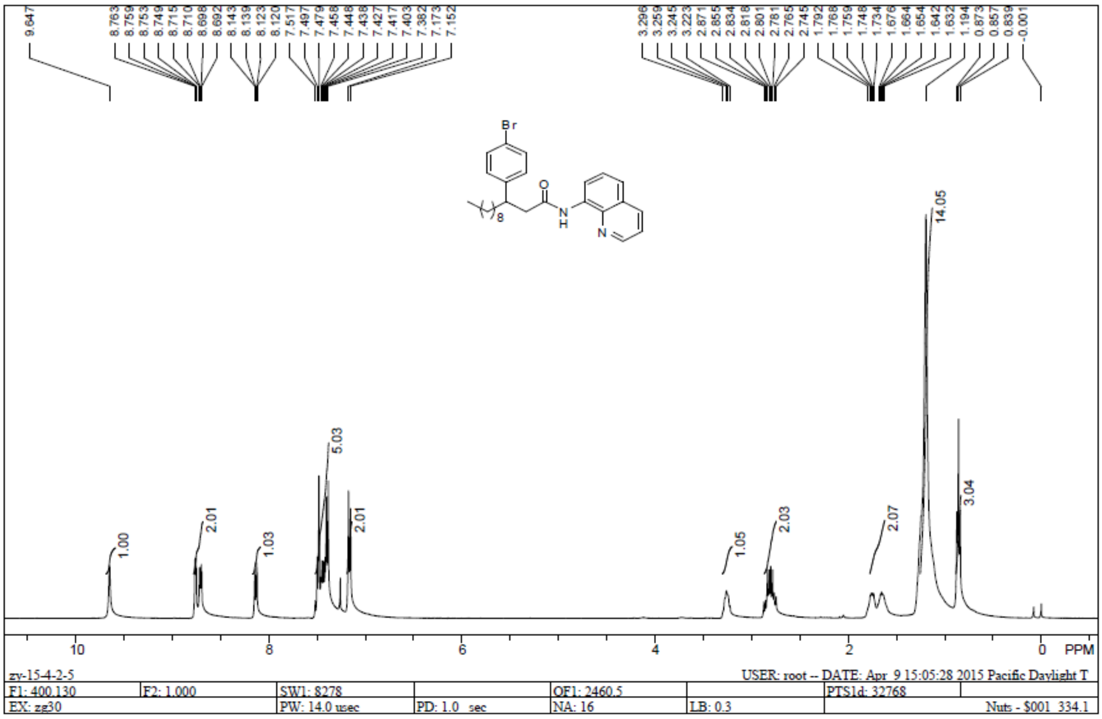

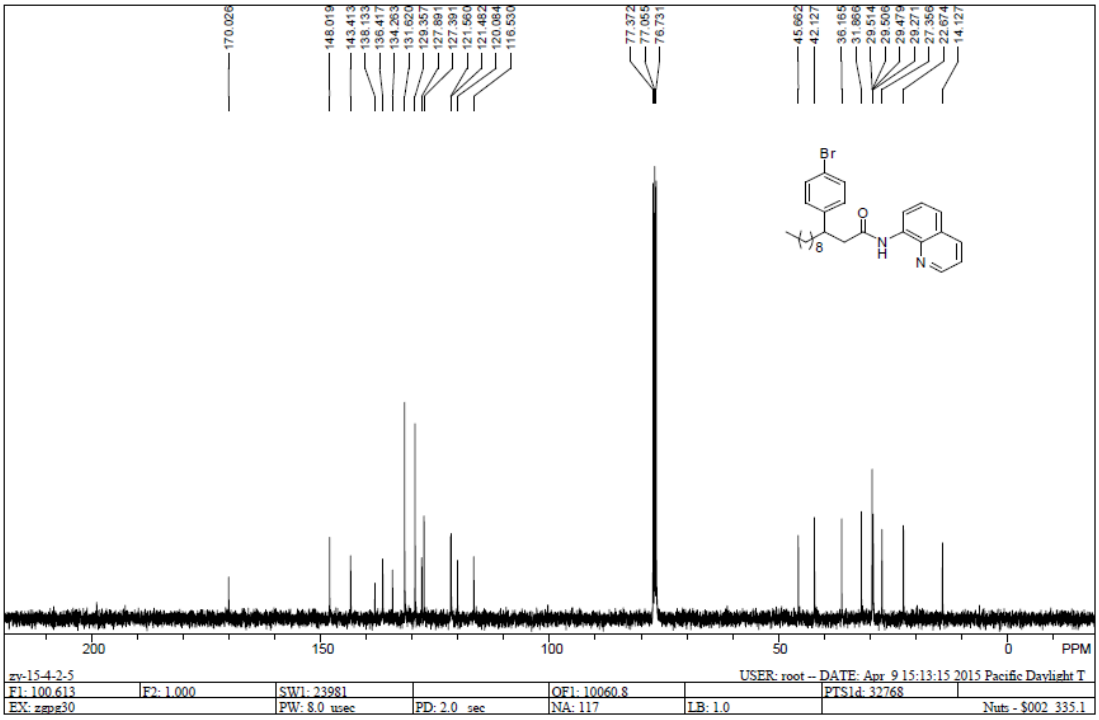

^{1}H and ^{13}C NMR of 4x

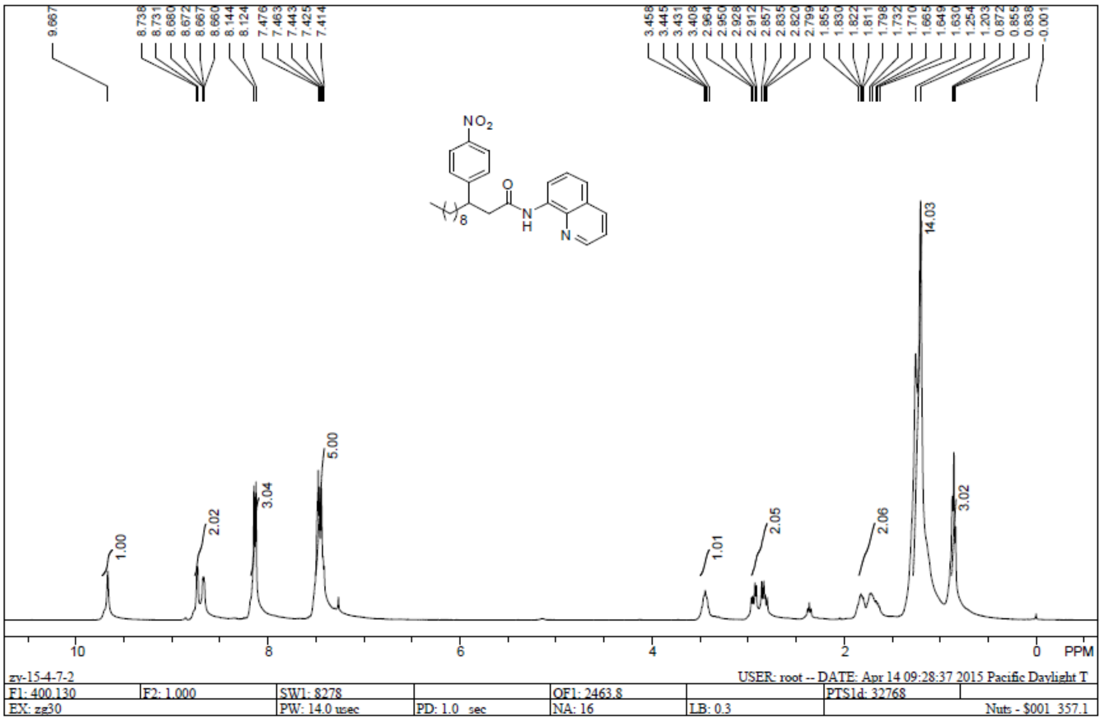

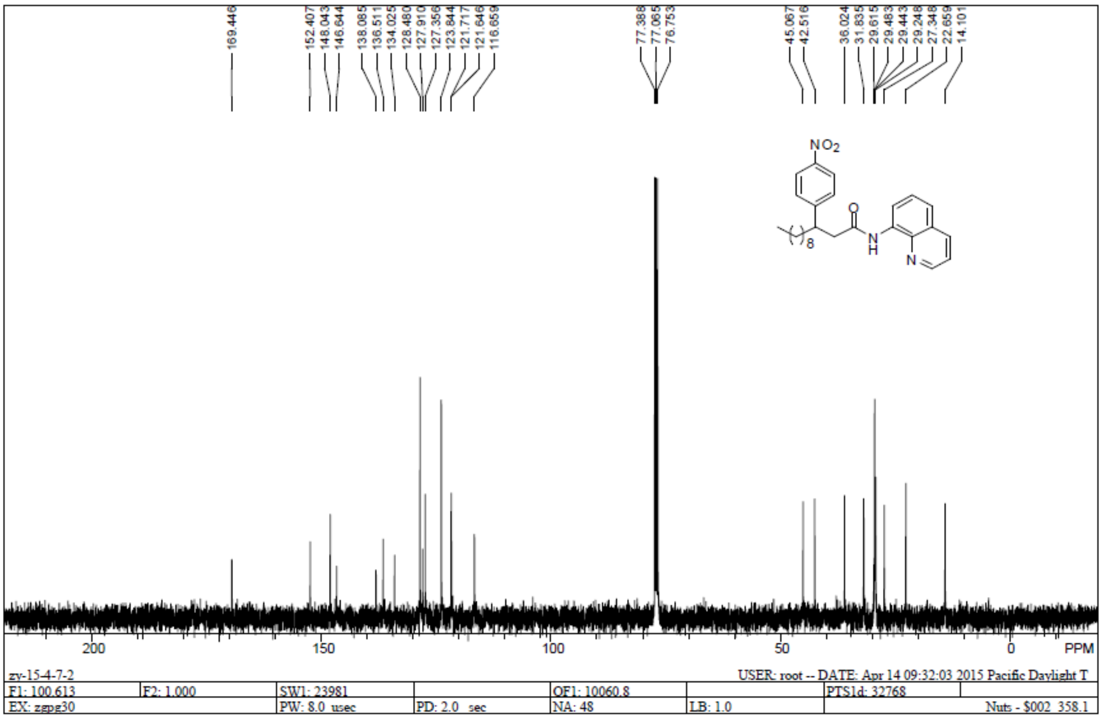

^{1}H and ^{13}C NMR spectra of 5a

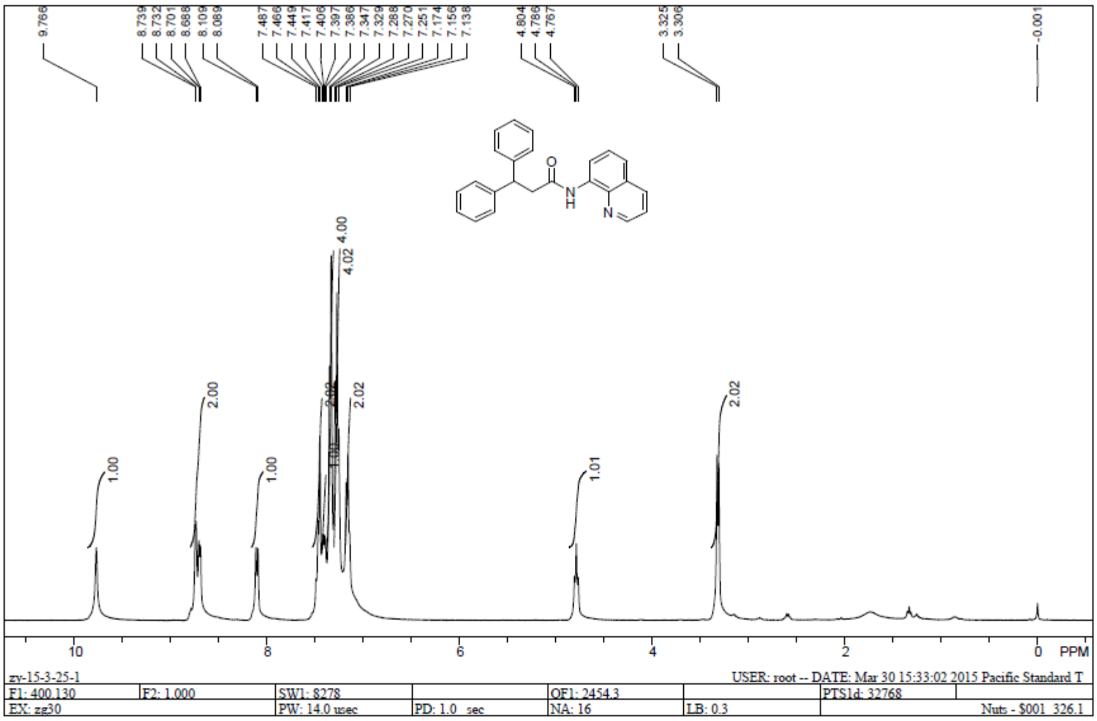

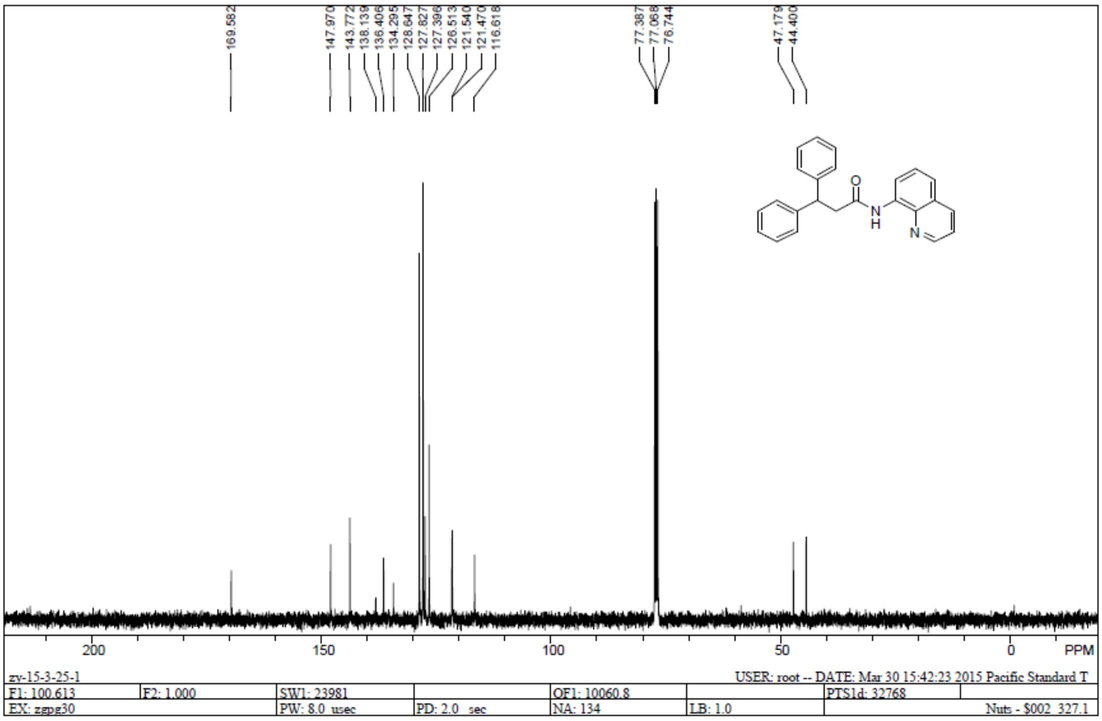

^{1}H and ^{13}C NMR spectra of 5b

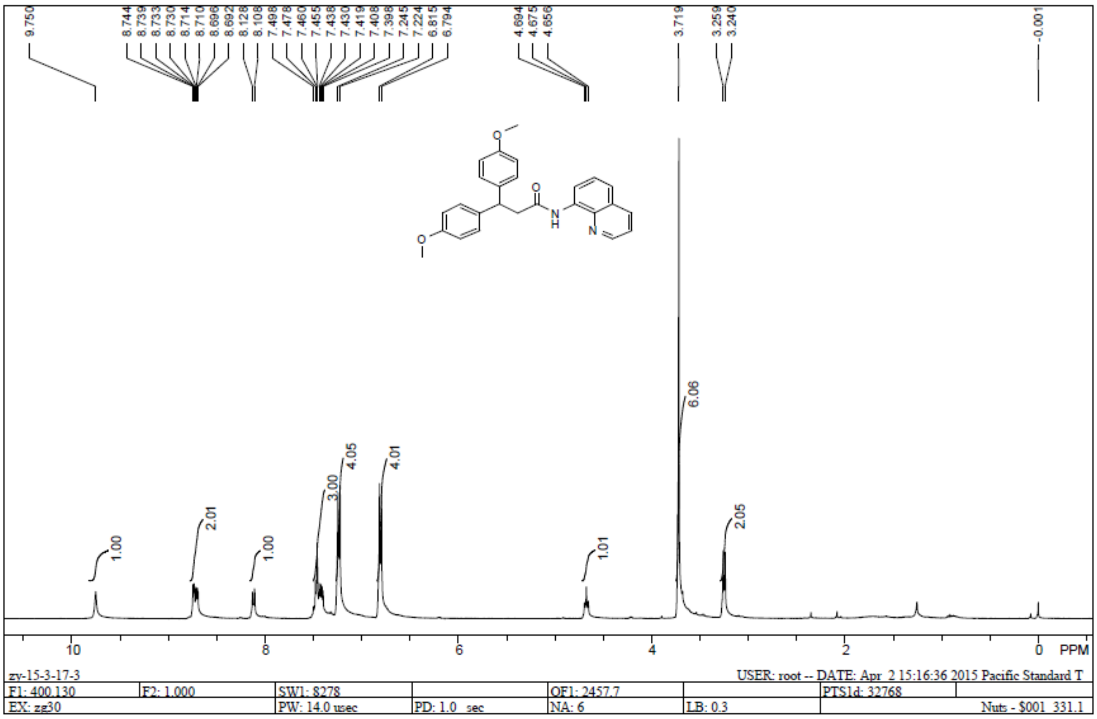

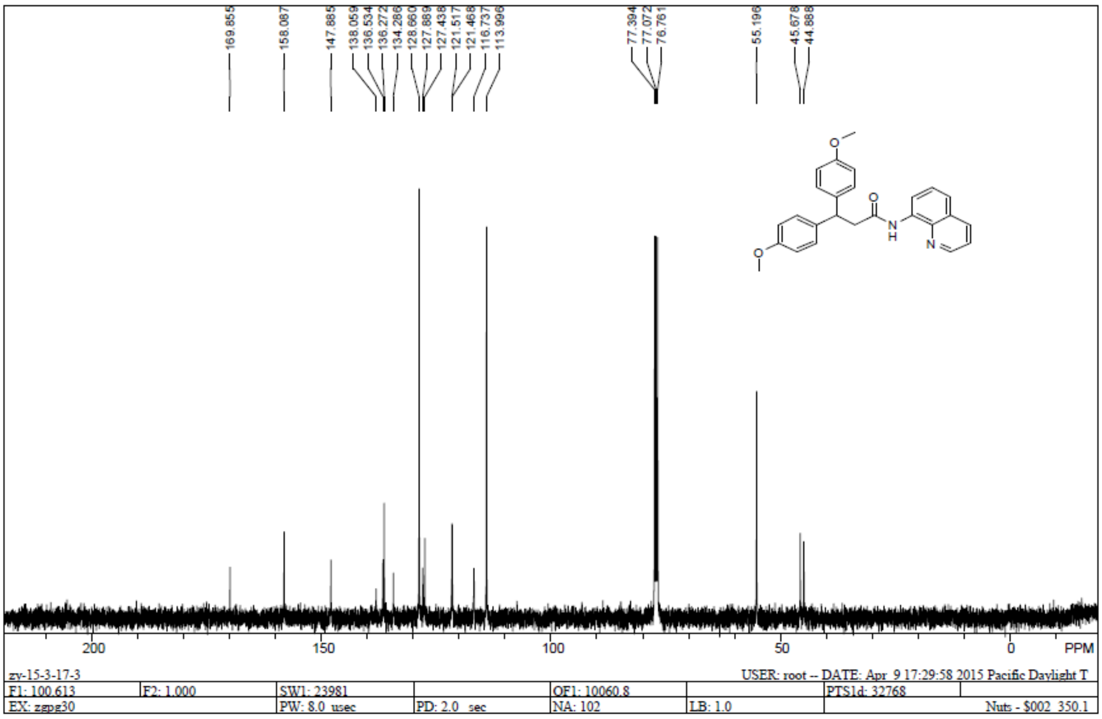

^{1}H and ^{13}C NMR spectra of 6a

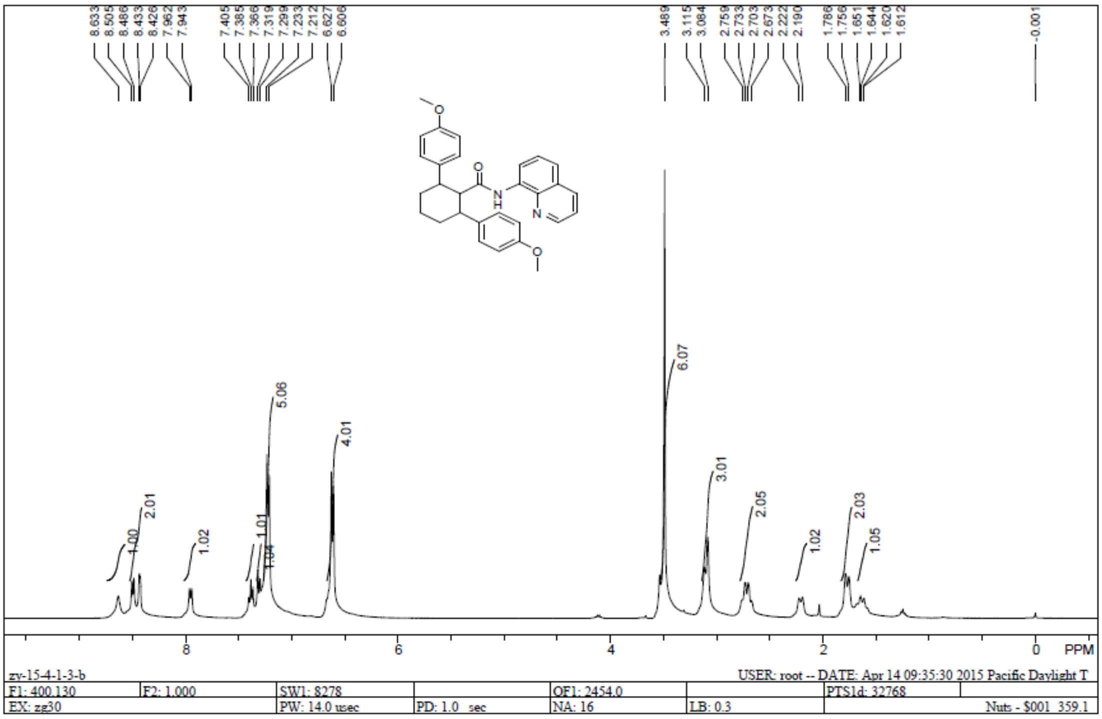

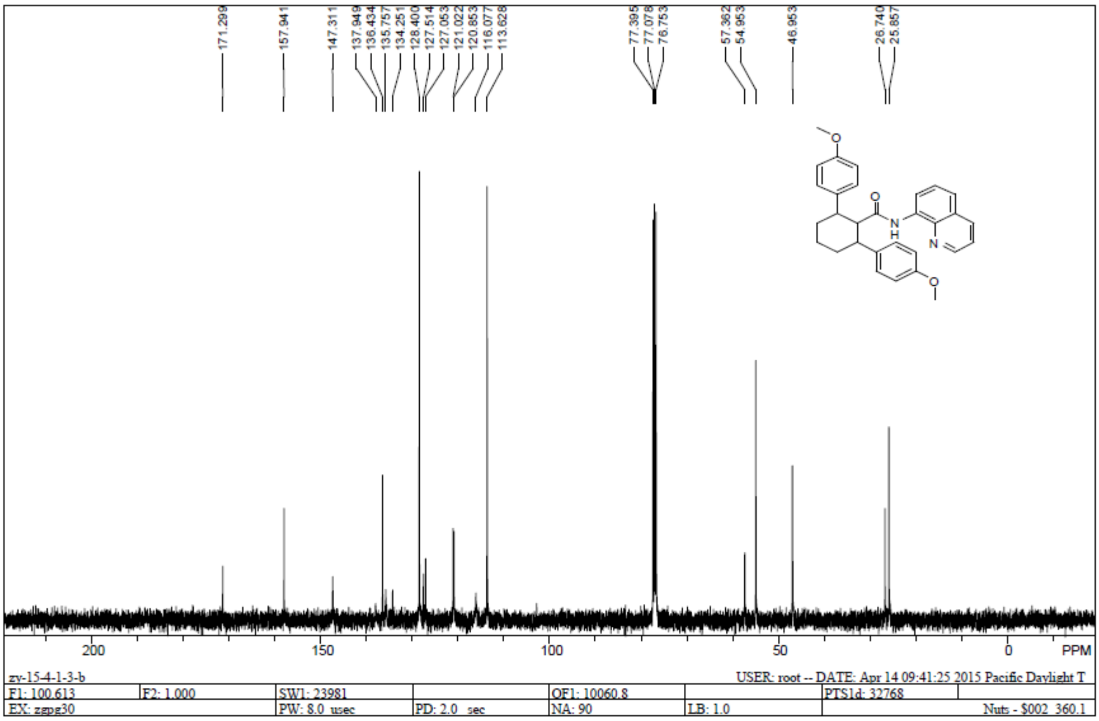

^{1}H and ^{13}C NMR of 6b

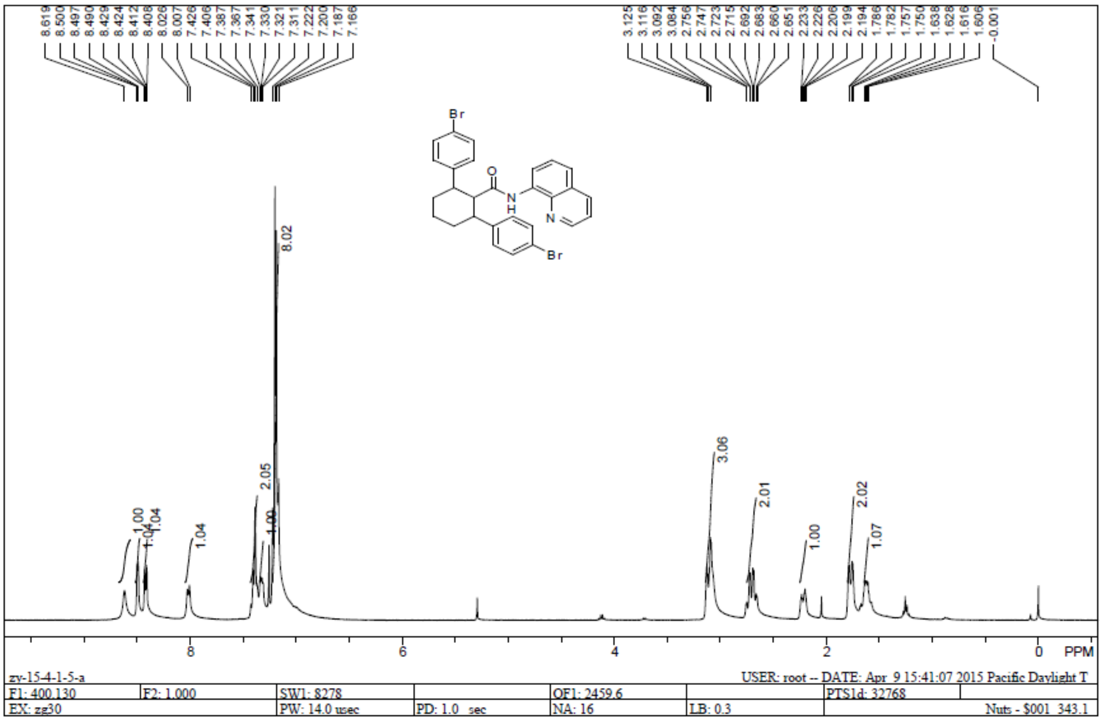

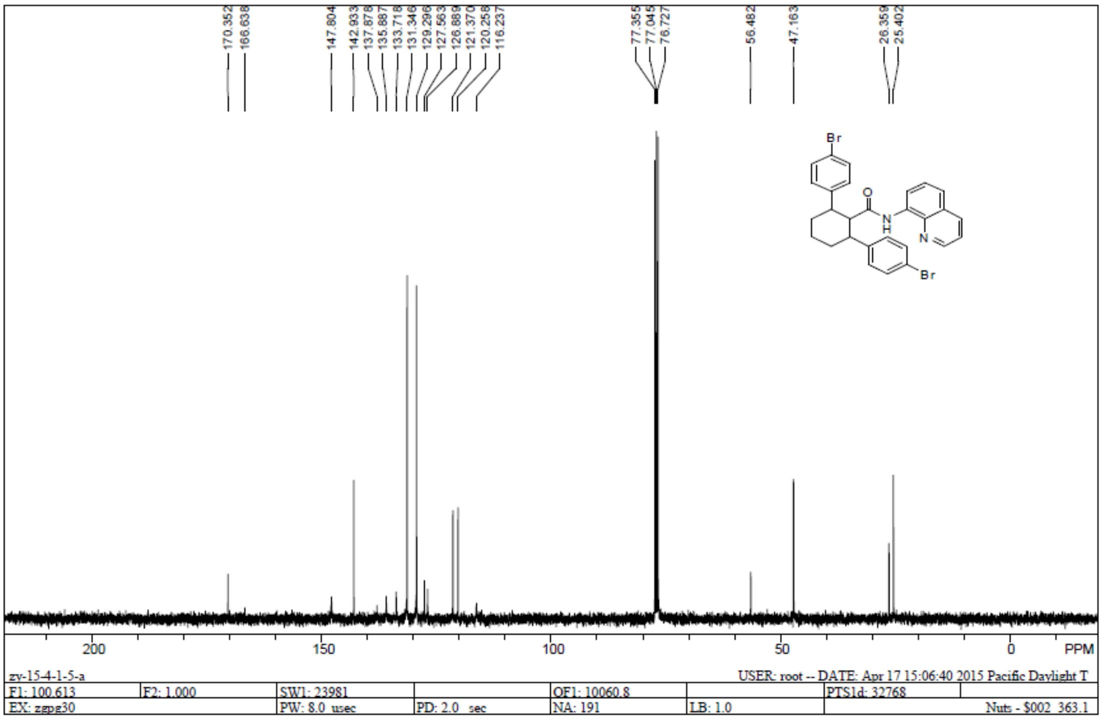

Supplement: File 1 — Experimental details on the synthesis of all products 4, 5 and 6; full characterization data as well as 1H/13C NMR spectra of all products. [file Beilstein_J_Org_Chem-12-1122-s001.pdf]
